# The impact of COVID-19 and bushfires on the mental health of Australian adolescents: a cross-sectional study

**DOI:** 10.1186/s13034-023-00583-1

**Published:** 2023-03-09

**Authors:** Joanne R. Beames, Kit Huckvale, Hiroko Fujimoto, Kate Maston, Philip J. Batterham, Alison L. Calear, Andrew Mackinnon, Aliza Werner-Seidler, Helen Christensen

**Affiliations:** 1 Black Dog Institute, University of New South Wales, Sydney, NSW Australia; 2 Centre for Digital Transformation of Health, University of Melbourne, Melbourne, VIC Australia; 3 Centre for Mental Health Research, Australian National University, Canberra, Australian Capital Territory Australia; 4 School of Psychiatry, Faculty of Medicine, University of New South Wales, Sydney, NSW Australia

**Keywords:** COVID-19, Bushfire, Disaster, Youth mental health, Adolescent

## Abstract

**Background:**

When COVID-19 spread to Australia in January 2020, many communities were already in a state of emergency from the Black Summer bushfires. Studies of adolescent mental health have typically focused on the effects of COVID-19 in isolation. Few studies have examined the impact of COVID-19 and other co-occurring disasters, such as the Black Summer bushfires in Australia, on adolescent mental health.

**Methods:**

We conducted a cross-sectional survey to examine the impact of COVID-19 and the Black Summer bushfires on the mental health of Australian adolescents. Participants (N = 5866; mean age 13.61 years) answered self-report questionnaires about COVID-19 diagnosis/quarantine (being diagnosed with and/or quarantined because of COVID-19) and personal exposure to bushfire harm (being physically injured, evacuated from home and/or having possessions destroyed). Validated standardised scales were used to assess depression, psychological distress, anxiety, insomnia, and suicidal ideation. Trauma related to COVID-19 and the bushfires was also assessed. The survey was completed in two large school-based cohorts between October 2020 and November 2021.

**Results:**

Exposure to COVID-19 diagnosis/quarantine was associated with increased probability of elevated trauma. Exposure to personal harm by the bushfires was associated with increased probability of elevated insomnia, suicidal ideation, and trauma. There were no interactive effects between disasters on adolescent mental health. Effects between personal risk factors and disasters were generally additive or sub-additive.

**Conclusions:**

Adolescent mental health responses to community-level disasters are multi-faceted. Complex psychosocial factors associated with mental ill health may be relevant irrespective of disaster. Future research is needed to investigate synergistic effects of disasters on young mental health.

**Supplementary Information:**

The online version contains supplementary material available at 10.1186/s13034-023-00583-1.

Converging evidence shows that adolescents can develop severe persistent psychological symptoms following nearly all types of natural disaster [1–5]. Commonly investigated outcomes include post-traumatic stress disorder (PTSD), depressive and anxiety disorders, disturbed behaviour, and impaired functioning [e.g., 3, 4, 6]. The role of cumulative early life adversities on stress is well documented [7–10], however psychological effects of co-occurring disasters on adolescent mental health have seldom been investigated. Amidst the widespread impact of COVID-19, young Australians have been simultaneously exposed to other natural disasters. A prime example is the 2019–2020 “Black Summer” bushfires, one of the worst on record for the country [11]. The nature of the relationship between COVID-19 and the Black Summer bushfires on adolescent mental health is yet to be fully understood. Direct tests of how these co-occurring disasters affect adolescent mental health are necessary to plan appropriate supports and facilitate disaster planning to optimise preventative efforts.

The psychological effects of COVID-19 and the Black Summer bushfires have been explored in adult samples. Two studies examined depression and anxiety symptoms during COVID-19 in a representative Australian adult cohort using cross-sectional [12] and longitudinal data [13]. Direct contact with COVID-19 had no/minimal effects on symptom levels [12, 13]. Bushfire exposure, defined through self-reports of being affected by fire or by smoke, was associated with decreased psychological wellbeing at the early acute phase of the COVID-19 pandemic [12] and increasing depression and anxiety symptoms several months later [13]. Another empirical study investigated the effects of these community threats on the mental health of a broad Australian sample that included adolescents [14]. Using phone-based experience-sampling methods, this study tracked 755 Australians, aged 13 years or older, between 2018 and 2020 [14]. Anxiety symptoms increased during COVID-19 but not during the bushfires, whereas depression increased during the bushfires and was sustained throughout COVID-19 [14]. Although providing much needed information about mental health trajectories, these studies did not specifically examine adolescents or interactive effects between COVID-19 and bushfires.

Adolescents tend to be more severely affected by disasters than adults [15, 16], and younger age is a pre-disaster risk factor for post-disaster mental illness [17]. Many factors can influence how adolescents respond to disasters, including characteristics of the disaster (e.g., severity) and the type/degree of exposure to it, individual characteristics, family factors, and the social environment [6, 9]. Some characteristics of COVID-19 and the bushfires experienced by young people are qualitatively different. For example, a critical indirect effect of COVID-19 on adolescent functioning and mental health was social isolation (especially from peers) and loneliness from mandated social distancing, lockdowns, and quarantines [18, 19]. Mandated social isolation is likely less of a concern in other community disasters such as bushfires. Concerns relevant to adolescents and their families unique to bushfires typically relate to proximity to the fire, destruction and loss of one’s home and belongings, being displaced, and physical injuries from smoke inhalation and fire [20–22]. These differences in disaster experiences may translate to different mental health responses and stress reactivity. At the individual level, prior exposure to disasters or trauma, mental illness history, female gender, other gender identities, and low household income are related to mental ill health following disasters [17, 23–26]. Further research is needed to delineate the interactions between personal risk factors and co-occurring disasters on adolescent mental health.

The theory of syndemics suggests that diseases (e.g., mental health conditions or symptoms) co-occur in temporal or geographical contexts due to harmful social conditions, and that they interact at the level of populations and individuals to produce mutually harmful outcomes [27–29]. Although the theory is widespread, syndemics is poorly understood and the extent to which there is empirical support for disease interactions in unclear [27, 29]. The joint effects of COVID-19 and the Black Summer bushfires on adolescent mental health are likely to be complex. For example, COVID-19 and the Black Summer bushfires might have mutually causal effects, wherein risk factors are reciprocal and differentially affect individuals who have experienced other adverse events. Alternatively, they might be synergistically interacting, whereby the disease burden attributable to joint health risks exceeds the sum of the disease burden of the health risks in isolation [28]. Exploring interactions between these disasters through the lens of syndemic theory is a novel contribution that will advance disaster research and practice [29]. Understanding how disasters (or different facets of disasters) interact to influence mental health symptoms will help to identify practical measures, including public policy, to provide support at the population level. Along with the theory of syndemics, the stress-diathesis model provides a framework for exploring interactions with personal risk factors. According to the model, stressors are events (e.g., Black Summer bushfires, COVID-19) that occur as a precursor to the onset of a mental health disorder. Diatheses are necessary precursors to the development of poor mental health and can include biological, environmental, or psycho-social risk factors [30]. Further research is needed to identify adolescents who are more vulnerable to developing mental health problems following multiple disasters.

## Current study

To the best of our knowledge, this is the first study to directly examine the interaction between exposure to the COVID-19 pandemic and the Black Summer bushfires on the mental health of Australian adolescents. In a cross-sectional retrospective study, our primary aim was to examine relationships between exposure to COVID-19 and bushfire personal harm on psychological distress, depression, anxiety, insomnia, suicidality, and post-traumatic stress symptoms while controlling for personal risk factors. Based on the literature, we controlled for gender, household income, mental illness history, and adverse childhood experiences [16, 17, 24, 25]. Given that minority groups may be particularly vulnerable due to stigmatisation, having fewer supports, and experiencing more barriers to accessing mental health care, we also controlled for sexual orientation and cultural and linguistic diversity. Building on prior literature [e.g., 31–34], we tested whether exposure to COVID-19 and the bushfires interacted to influence adolescent mental health. Consistent with the stress-diathesis model [35], our secondary aim was to identify whether theoretically informed personal risk factors (diatheses) moderated the effects of these adverse environmental events on mental health. We were particularly interested in identifying whether interactions, if present, were synergistic or additive/sub-additive.

## Methods

Data were collected as part of the Future Proofing Study. Future Proofing is a large-scale, 5-year, cluster randomised controlled trial investigating whether psychological therapies delivered by smartphone application can prevent depression in adolescents. This registered trial (ACTRN12619000855123) has ethical approval from the University of New South Wales Human Research Ethics Committee (HC180836), NSW Government State Education Research Applications Process Approval (SERAP 2019201) and relevant Catholic Schools Dioceses across Australia. Full details about the trial are detailed in the protocol paper and cohort profile paper [36, 37]. Data reported in the current paper were part of baseline measurement of the trial collected in two cohorts between August 2020 and November 2021. This sample is a subset of the larger Future Proofing cohort, excluding participants who were recruited pre-COVID-19. The Black Summer bushfire season occurred before data collection commenced, with exposure to bushfires collected retrospectively; varying levels of COVID-19 restrictions coincided with the entire data collection period.

### Diatheses (personal risk factors)

Information was collected on participants’ gender identification, sexual orientation, perceived household wealth, and language spoken most at home. Participants were also asked to indicate whether they had ever been diagnosed with a range of mental illnesses (e.g., major depression, social anxiety disorder, generalised anxiety disorder, panic disorder) by a professional, and to complete the Adapted Behavioural Risk Factor Surveillance System Adverse Childhood Experience Module (BRFSS-ACE). The BRFSS-ACE (e.g., Have you ever felt like your life was in serious danger or that you would be harmed?; Have you ever been in out-of-home or foster care?) is a widely used 8-item scale to assess adverse childhood experiences and has acceptable psychometric properties [38]. For both mental illness history and adverse childhood experiences, we calculated a composite score from item responses, which was then dichotomised to identify participants who had no mental illness history/adverse experiences and those who had a mental illness history/adverse experiences.

### Stressors

#### COVID-19 diagnosis and/or quarantine

Participants were asked to complete a set of 26 questions generated from previous COVID-19 surveys with adults and adolescents [19, 39]. Topics included COVID-19 testing, diagnosis, and quarantine, physical/mental health, perceived risk about catching COVID-19, and impact on school and education, friends, family, and emotion/mental health. We calculated a composite score of items assessing personal impact of COVID-19, including whether participants had been diagnosed and/or required to quarantine for 14 days. Responses were dichotomised to identify individuals who did not receive a diagnosis nor have to quarantine and those who did (at least one).

#### Exposure to bushfire personal harm

Participants were asked a Yes/No question about whether they were affected by the 2019–2020 bushfires in any way. Nine Yes/No questions about living/schooling area (gating question), trauma threats (e.g., evacuation from home/school), uncontrollable events (e.g., home/possessions damaged by fire), and subjective emotional response to the fires were also adapted from a survey developed to assess exposure to bushfires in adults [40]. We calculated a composite score of items assessing exposure to bushfire personal harm, including evacuation from home or school, damage or destruction of own home/possessions, and injuries to self. This approach was informed by adult research showing that more severe mental health outcomes are associated with certain characteristics of environmental events (e.g., physical injury, threats to life, property loss; [40, 41]). Responses were dichotomised to represent no exposure to bushfire personal harm and exposure to bushfire personal harm (i.e., endorsement of one or more harms).

### Mental health measures

Participants completed measures of depressive symptoms (Patient Health Questionnaire-Adolescent Version; [42]), psychological distress (Distress Questionnaire-5; [43, 44]), anxiety (Children’s Anxiety Scale Short-Form; [45]), insomnia (Insomnia Severity Index; [46]), suicidal ideation (Suicidal Ideation Attributes Scale; [47]), and two modified versions of the Child Traumatic Stress Questionnaire [48], one to assess bushfire trauma and the other to assess COVID-19 trauma. Only participants who lived or went to school in an area threatened by the bushfires were asked to complete the bushfire CTSQ. All measures have sound psychometric properties and have been used in youth samples. All measures were dichotomised into cut-off thresholds for elevated symptoms [43, 46–49]. Given that rates of common mental health problems are typically high in adolescents, and the concern of over-reporting through self-report versus clinical diagnosis, specificity was prioritised over sensitivity (see Table 1).

**Table 1:** Applied cut-off thresholds for each mental health measure

Mental health measure	Cut-off thresholds
Psychological distress	≤ 13 normal range; ≥ 14 elevated range
Depression	≤ 14 normal range; ≥ 15 elevated range
Anxiety	≤ 13 normal range; ≥ 14 elevated range
Insomnia	≤ 14 normal range; ≥ 15 elevated range
Suicidal ideation	≤ 20 normal range; ≥ 21 elevated range
Trauma	≤ 4 normal range; 5 elevated range

### Procedure

Parents and students provided active informed consent for students to take part in the study. Students completed baseline assessment during a session that was facilitated by the research team from Black Dog Institute either in person at school or remotely via Zoom. The session typically lasted between 1–2 h. Students accessed the baseline trial questionnaire via a secure online portal and were encouraged to complete the questionnaire on a smartphone, laptop, tablet, or desktop computer. The questionnaire took up to 45-min to complete. See the cohort profile for additional details [37].

### Statistical analyses

Data preparation and descriptive/frequency statistics were conducted in SPSS v. 25. Binomial Generalised Linear Mixed Models (GLMMs) using the logit link function were estimated in R v 2021.02.1 [50] with the lme4 package [51]. The estimation method was maximum likelihood (adaptive Gaussian quadrature rule approximation). Participants with missing data were removed from the models under the assumption that data were missing at random. GLMMs were appropriate given our binary dependent variables and hierarchical data structure.

Binomial GLMMs were estimated to test the effects of personal COVID-19 diagnosis/quarantine (0 = no, 1 = yes), personal harm from bushfire (0 = no, 1 = yes), and personal risk factors on mental health (psychological distress, depression, anxiety, insomnia, suicidal ideation, trauma; 0 = normal range, 1 = elevated range). Personal risk factors included adverse childhood experiences, mental illness history, gender identification, sexual orientation, perceived household wealth, and language spoken most at home. The adverse childhood experiences measure was dichotomised into no (0) and yes (1); mental illness history was dichotomised into no (0) and yes (1); Gender was categorised into male (0), female (1), other (2), and prefer not to say (3); sexual orientation was categorised into heterosexual or straight (0), sexuality diverse (1), unsure (2), and prefer not to say (3); perceived household wealth was categorised into high (0), low (1), and prefer not to say (2); language spoken at most home was dichotomised into English (0) and other (1). Prefer not to say response options were included to minimise missing data; they were not interpreted in significant comparisons.

#### Model specification

To address the primary aim, personal risk factors (adverse childhood experiences, mental illness history, gender, sexual orientation, perceived household wealth,, language spoken most at home) were entered simultaneously as fixed effects into model 1. School was included as a random effect in the models to reflect the clustered sampling of students within schools. COVID-19 diagnosis/quarantine (model 2), bushfire personal harm (model 3), and the COVID-19 diagnosis/quarantine × bushfire personal harm interaction (model 4) were then sequentially added into the models as fixed effects. Likelihood Ratio Tests were used to compare nested mixed models, and to test the random effect of school in the best fitting model (i.e., by comparing to the fixed effect model). The impact of approximate Gaussian quadrature (AGQ) points on parameter estimates and log-likelihood value at convergence were also checked for the best-fitting models. There were virtually no differences between examined quadrature points (AGQ = 11, AGQ = 15, and AGQ = 25), indicating model stability. Models estimated with 11 quadrature points are reported in the Results section below. This process was repeated for each mental health measure (e.g., psychological distress, depression etc.). Statistical assumptions of the best-fitting models were checked using DHARMa [52] and performance [53] packages. Marginal effects were estimated as predicted probabilities from the best fitting models. A Bonferroni correction was applied, resulting in a model-wise significance threshold of *p* < 0.007 (*k* = 7).

To address the secondary aim, personal risk factors, COVID-19 diagnosis/quarantine, and bushfire personal harm were simultaneously entered as fixed effects into model 1 (main effects model). School was also added as a random effect. Two-way interactions between the disaster variables and risk factors (adverse childhood experiences, mental illness history, gender, sexual orientation) were then simultaneously added into model 2. Moderation by each risk factor (e.g., gender) was modelled separately for each mental health measure. These two-way interactions were the effects of interest; they are a direct test of the stress-diathesis model, whereby risk factors are considered types of diatheses. Likelihood Ratio Tests were used to compare nested mixed models. Simple effects were explored to interpret significant two-way interactions. Model assumptions and significance were checked as outlined above.

#### Model assumptions

Exploration of scaled residuals indicated that the assumptions for binomial GLMMs were generally met. Scaled residuals followed the expected distribution (Kolmogorov–Smirnov test: *p*s > 0.18) and there was no evidence for significant dispersion (*ps* > 0.73) or outliers (± 3*SD*). Multicollinearity was low, except for some models including interaction terms. Random effects were normally distributed. All models converged.

## Results

### Descriptive analysis

#### Sample characteristics

The sample consisted of 5866 adolescents (*n* = 1911, 32.6% in 2020; *n* = 3955, 67.4% in 2021), with a mean age of 13.91 years (*SD* = 0.52). Cohorts were conceptually similar (see Additional file 1: Tables S1, S2 in the Supplementary Materials) and, as such, all data were analysed together. Most adolescents self-identified their current gender as female (*n* = 2925, 49.9%) or male (*n* = 2662, 45.4%), and their sexual orientation as heterosexual (*n* = 4101, 72.7%). For those that reported perceived household wealth, there was a roughly even split between low (*n* = 2439, 41.6%) and high (*n* = 2596, 44.3%). Most adolescents indicated that they were not Aboriginal and/or Torres Strait Islander (*n* = 5404, 92.1%) and that English was their primary language spoken at home (*n* = 5481, 93.5%). Further, 17.8% (*n* = 1046) reported being diagnosed with a mental illness by a professional and 68.7% (*n* = 4028) reported exposure to one more or more adverse childhood experiences. Participant schools were largely located in major city areas (*n* = 4334, 73.9%) in New South Wales and Victoria (*n* = 5398, 92%), with a roughly even proportion from the government (*n* = 3135, 53.4%) sector and the independent sector (*n* = 2731, 46.6%).

#### Stressors

##### COVID-19 diagnosis/quarantine

Of the participants, 802 (13.7%) endorsed receiving a COVID-19 diagnosis and/or having to quarantine. There were no significant differences between state (χ(4)^2^ = 1.80, *p* = 0.77; see Additional file 1: Tables S3, S4 in the Supplementary Materials).

##### Exposure to bushfire personal harm

Most participants (*n* = 3758, 66.6%) reported that they were not affected in any way by the 2019–2020 bushfires and that they did not live or go to school in an area that came under threat (*n* = 4891, 84.5%). Of those participants who were affected, 384 (6.5%) endorsed exposure to at least one bushfire personal harm (i.e., being evacuated, home or possessions damaged/destroyed, personal injury). There was variation in reported bushfire exposure between states, χ(4)^2^ = 11.70, *p* = 0.02, with exposure more frequent in New South Wales (see Additional file 1: Tables S3, S4). Also see the Supplementary Materials for additional descriptive statistics about bushfire exposure and impact.

#### Mental health

Almost one-third (*n* = 1894, 32.3%) of the sample reported elevated psychological distress and 15.7% (*n* = 920) reported elevated depressive symptoms. For anxiety, insomnia, and suicidal ideation, 19.1% (*n* = 1121), 11.5% (*n* = 671), and 5.1% (*n* = 274) reported elevated symptoms, respectively. Approximately one-fifth of the sample reported elevated trauma symptoms related to COVID-19 (*n* = 1046, 18.2%) and the bushfires (*n* = 196, 21.8%). See Additional file 1: Table S5 in the Supplementary Materials for the proportion of participants scoring within the normal and elevated ranges for each mental health measure. Also see Table 2 for a descriptive comparison of sample characteristics, including mental health measures, as a function of COVID-19 diagnosis/quarantine and bushfire personal harm.

**Table 2:** Descriptive comparison of sample characteristics by COVID-19 diagnosis/quarantine and bushfire personal harm

Sample Characteristics	COVID-19 Diagnosis/Quarantine		Bushfire Personal Harm	
	No	Yes	No	Yes
Adverse childhood experiences (n, %)				
No	1646 (90.1)	181 (9.9)	1739 (95.2)	88 (4.8)
Yes	3408 (84.6)	620 (15.4)	3732 (92.7)	296 (7.3)
Prefer not to say	10 (90.9)	1 (9.1)	11 (100)	0 (0)
Mental illness history (n, %)				
No	4199 (87.1)	621 (12.9)	4525 (93.9)	295 (6.1)
Yes	865 (82.7)	181 (17.3)	957 (91.5)	89 (8.5)
Gender identification (n, %)				
Male	2320 (87.2)	342 (12.8)	2496 (93.8)	166 (6.2)
Female	2521 (86.2)	404 (13.8)	2729 (93.3)	196 (6.7)
Other	141 (77.9)	40 (22.1)	164 (90.6)	17 (9.4)
Prefer not to say	82 (83.7)	16 (16.3)	93 (94.9)	5 (5.1)
Sexual orientation (n, %)				
Heterosexual or straight	3564 (86.9)	537 (13.1)	3830 (93.4)	271 (6.6)
Sexuality diverse	605 (83.1)	123 (16.9)	676 (92.9)	52 (7.1)
Unsure	428 (84.9)	76 (15.1)	471 (93.5)	33 (6.5)
Prefer not to say	236 (84.3)	44 (15.7)	263 (93.9)	17 (6.1)
Perceived household wealth (n, %)				
High	2257 (86.9)	339 (13.1)	2442 (94.1)	154 (5.9)
Low	2075 (85.1)	364 (14.9)	2265 (92.9)	174 (7.1)
Prefer not to say	732 (88.1)	99 (11.9)	775 (93.3)	56 (6.7)
Language spoken most at home (n, %)				
English	4745 (86.6)	736 (13.4)	5114 (93.3)	367 (6.7)
Other	319 (83.1)	65 (16.9)	367 (95.6)	17 (4.4)
Psychological distress (n, %)				
Normal range	3491 (88)	477 (12)	3723 (93.8)	245 (6.2)
Elevated range	1569 (82.8)	325 (17.2)	1755 (92.7)	139 (7.3)
Depression (n, %)				
Normal range	4317 (87.3)	628 (12.7)	4648 (94)	297 (6)
Elevated range	746 (81.1)	174 (18.9)	833 (90.5)	87 (9.5)
Anxiety (n, %)				
Normal range	4137 (87.3)	600 (12.7)	4457 (94.1)	280 (5.9)
Elevated range	919 (82)	202 (18)	1017 (90.7)	104 (9.3)
Insomnia (n, %)				
Normal range	4501 (86.9)	676 (13.1)	4864 (94)	313 n
Elevated range	545 (81.2)	126 (18.8)	600 (89.4)	71 (10.6)
Suicidal ideation (n, %)				
Normal range	4458 (87.2)	656 (12.8)	4796 (93.8)	318 (6.2)
Elevated range	219 (79.9)	55 (20.1)	240 (87.6)	34 (12.4)
COVID-19 trauma (n, %)				
Normal range	593 (84.4)	110 (15.6)	434 (61.7)	269 (38.3)
Elevated range	148 (75.5)	48 (24.5)	81 (41.3)	115 (58.7)
Bushfire trauma (n, %)				
Normal range	593 (84.4)	110 (15.6)	434 (61.7)	269 (38.3)
Elevated range	148 (75.5)	48 (24.5)	81 (41.3)	115 (58.7)

### Primary analyses

Modelling results and Likelihood Ratio Tests comparing nested models for psychological distress, depression, anxiety, insomnia, suicidal ideation, and COVID-19 and bushfire trauma are presented in Additional file 1: Tables S6–S12 in the Supplementary Materials. For psychological distress and COVID-19 trauma, the best-fitting model included personal risk factors and COVID-19 diagnosis/quarantine (*χ*(1)^2^ ≥ 5.48, *ps* ≤ 0.02). For the remaining mental health measures, the best fitting model included personal risk factors, COVID-19 diagnosis/quarantine, and bushfire personal harm (*χ*^*2*^ ≥ 4.72, *p*s ≤ 0.03). The interaction between COVID-19 diagnosis/quarantine and bushfire personal harm did not explain a significant amount of variance for any mental health variable (*χ*^2^ ≤ 1.40, *p*s ≥ 0.24). Comparison of best-fitting models with and without the random effect of school indicated that school explained a significant proportion of variance in psychological distress, depression, anxiety, insomnia, and suicidal ideation, but not the trauma-related variables (see Additional file 1: Tables S6–S12). School was retained in all models given the study design. However, the intra-class correlation coefficients were relatively small (range = 0.04–0.13), indicating that minimal heterogeneity in observations was attributable to school. For example, 33% of the total variance in psychological distress was explained by fixed effects (marginal *R*^*2*^ = 0.33); only 1% of the total variance was explained by the random effect of school (conditional *R*^*2*^ = 0.34). Predicted probabilities and pairwise comparisons of explanatory variables for the best-fitting GLMMs are presented in Tables 3, 4 and are interpreted below.

**Table 3:** Predicted probabilities (standard error) for the best-fitting mixed models for each dependent variable

Explanatory Variables	Psychological distress	Depression	Anxiety	Insomnia	Suicidal ideation	COVID-19 trauma	Bushfire trauma
Adverse childhood experiences							
No (ref)	0.41 (0.03)	0.17 (0.03)	0.22 (0.03)	0.16 (0.02)	0.03 (0.01)	0.20 (0.02)	0.30 (0.07)
Yes	0.69 (0.03)	0.42 (0.03)	0.40 (0.03)	0.27 (0.03)	0.15 (0.03)	0.34 (0.03)	0.39 (0.08)
Mental illness history							
No (ref)	0.40 (0.03)	0.18 (0.02)	0.19 (0.02)	0.14 (0.02)	0.04 (0.01)	0.21 (0.02)	0.29 (0.06)
Yes	0.69 (0.03)	0.41 (0.04)	0.44 (0.04)	0.29 (0.04)	0.12 (0.03)	0.32 (0.03)	0.40 (0.08)
Gender							
Male (ref)	0.26 (0.02)	0.14 (0.02)	0.11 (0.01)	0.10 (0.01)	0.04 (0.01)	0.15 (0.02)	0.23 (0.05)
Female	0.56 (0.02)	0.27 (0.03)	0.36 (0.03)	0.20 (0.02)	0.08 (0.02)	0.30 (0.03)	0.38 (0.07)
Other	0.74 (0.04)	0.52 (0.06)	0.50 (0.05)	0.35 (0.05)	0.18 (0.05)	0.36 (0.04)	0.31 (0.10)
Prefer not to say	0.64 (0.06)	0.25 (0.05)	0.34 (0.06)	0.25 (0.05)	0.04 (0.02)	0.26 (0.05)	0.47 (0.16)
Sexual orientation							
Heterosexual (ref)	0.43 (0.03)	0.18 (0.02)	0.22 (0.03)	0.16 (0.02)	0.04 (0.01)	0.21 (0.02)	0.24 (0.06)
Sexuality diverse	0.71 (0.03)	0.45 (0.04)	0.41 (0.04)	0.30 (0.03)	0.12 (0.03)	0.34 (0.03)	0.50 (0.08)
Unsure	0.54 (0.04)	0.24 (0.03)	0.27 (0.04)	0.18 (0.04)	0.05 (0.02)	0.22 (0.03)	0.28 (0.08)
Prefer not to say	0.52 (0.04)	0.28 (0.04)	0.31 (0.04)	0.22 (0.04)	0.08 (0.03)	0.28 (0.04)	0.38 (0.11)
Perceived household wealth							
High (ref)	0.51 (0.03)	0.23 (0.03)	0.26 (0.03)	0.18 (0.02)	0.06 (0.02)	0.24 (0.02)	0.30 (0.07)
Low	0.60 (0.03)	0.30 (0.03)	0.34 (0.03)	0.24 (0.03)	0.08 (0.02)	0.28 (0.02)	0.34 (0.07)
Prefer not to say	0.55 (0.03)	0.30 (0.04)	0.31 (0.04)	0.21 (0.03)	0.07 (0.02)	0.26 (0.03)	0.38 (0.08)
Language spoken most at home							
English (ref)	0.53 (0.03)	0.26 (0.03)	0.33 (0.03)	0.21 (0.02)	0.06 (0.01)	0.24 (0.02)	0.32 (0.05)
Other	0.57 (0.04)	0.29 (0.04)	0.27 (0.04)	0.21 (0.04)	0.07 (0.02)	0.28 (0.04)	0.36 (0.11)
COVID-19 diagnosis/quarantine							
No (ref)	0.52 (0.03)	0.26 (0.03)	0.28 (0.03)	0.19 (0.02)	0.06 (0.02)	0.22 (0.02)	0.31 (0.06)
Yes	0.58 (0.03)	0.30 (0.04)	0.32 (0.04)	0.23 (0.03)	0.08 (0.02)	0.31 (0.03)	0.38 (0.08)
Bushfire personal harm							
No (ref)	N/A	0.24 (0.02)	0.26 (0.03)	0.17 (0.02)	0.05 (0.01)	N/A	0.25 (0.06)
Yes	N/A	0.31 (0.04)	0.34 (0.04)	0.25 (0.04)	0.09 (0.03)	N/A	0.44 (0.08)

*Ref* reference category

**Table 4:** Pairwise comparisons for the best-fitting mixed models for each dependent variable

Pairwise comparison	Psychological Distress	Depression	Anxiety	Insomnia	Suicidal Ideation	COVID-19 Trauma	Bushfire Trauma
	OR (95% CI)	OR (95% CI)	OR (95% CI)	OR (95% CI)	OR (95% CI)	OR (95% CI)	OR (95% CI)
Adverse childhood experiences							
No (ref) vs yes	3.21 (2.73, 3.78)^*^	3.62 (2.83, 4.63)^*^	2.38 (1.96, 2.90)^*^	2.01 (1.59, 2.56)^*^	6.27 (3.54, 11.10)^*^	2.09 (1.74, 2.51)^*^	1.45 (0.92, 2.29)
Mental illness history							
No (ref) vs yes	3.38 (2.88, 3.98)^*^	3.22 (2.69, 3.85)^*^	3.28 (2.76, 3.89)^*^	2.47 (2.04, 3.00)^*^	3.31 (2.51, 4.37)^*^	1.77 (1.49, 2.09)^*^	1.58 (1.07, 2.34)
Gender							
Male (ref) vs female	3.65 (3.01, 4.43)^*^	2.41 (1.87, 3.10)^*^	4.85 (3.76, 6.24)^*^	2.36 (1.77, 3.13)^*^	2.20 (1.43, 3.41)^*^	2.44 (1.96, 3.03)^*^	2.07 (1.26, 3.40)^*^
Male (ref) vs other	8.30 (4.64, 14.85)^*^	6.97 (4.10, 11.84)^*^	8.33 (4.95, 14.01)^*^	4.97 (2.90, 8.50)^*^	5.85 (2.86, 12.00)^*^	3.10 (1.90, 5.07)^*^	1.48 (0.45, 4.81)
Female (ref) vs other	2.27 (1.29, 4.02)^*^	2.89 (1.76, 4.77)^*^	1.72 (1.06, 2.79)	2.11 (1.23, 3.45)^*^	2.66 (1.41, 4.99)*	1.27 (0.80, 2.03)	0.71 (0.22, 2.24)
Sexual orientation							
Heterosexual (ref) vs sexuality diverse	3.30 (2.53, 4.30)^*^	3.77 (2.84, 4.99)^*^	2.40 (1.82, 3.17)^*^	2.33 (1.71, 3.19)^*^	3.53 (2.28, 5.49)^*^	1.97 (1.50, 2.57)^*^	3.10 (1.56, 5.99)^*^
Heterosexual (ref) vs unsure	1.59 (1.91, 2.10)^*^	1.45 (1.01, 2.08)	1.30 (0.93, 1.82)	1.17 (0.78, 1.77)	1.40 (0.74, 2.66)	1.08 (0.78, 1.51)	1.18 (0.53, 2.60)
Sexuality diverse (ref) vs unsure	0.48 (0.34, 0.69)^*^	0.38 (0.26, 0.58)^*^	0.54 (0.37, 0.80)^*^	0.50 (0.32, 0.79)^*^	0.40 (0.20, 0.78)^*^	0.55 (0.38, 0.81)^*^	0.39 (0.15, 0.98)
Perceived household wealth							
High (ref) vs low	1.41 (1.20, 1.67)^*^	1.46 (1.18, 1.81)^*^	1.46 (1.20, 1.78)^*^	1.47 (1.16, 1.85)^*^	1.36 (0.96, 1.92)	1.29 (1.07, 1.55)^*^	1.18 (0.75, 1.85)
Language spoken most at home							
English (ref) vs other	1.18 (0.91, 1.54)	1.16 (0.83, 1.61)	0.76 (0.54, 1.05)	0.95 (0.65, 1.39)	1.20 (0.69, 2.07)	1.25 (0.94, 1.65)	1.22 (0.53, 2.79)
COVID-19 diagnosis/quarantine							
No (ref) vs yes	1.25 (1.04, 1.50)	1.25 (1.00, 1.55)	1.22 (0.99, 1.50)	1.24 (0.98, 1.56)	1.30 (0.92, 1.84)	1.55 (1.28, 1.87)^*^	1.40 (0.92, 2.15)
Bushfire personal harm							
No (ref) vs yes	N/A	1.40 (1.04, 1.88)	1.45 (1.09, 1.92)	1.60 (1.18, 2.19)^*^	1.95 (1.27, 3.00)^*^	N/A	2.36 (1.67, 3.34)^*^

“Prefer not to say” responses were not included in pairwise comparisons

*OR* odds ratio, *95% CI* 95% confidence intervals, *ref* reference category

^*^p < .007

Subgroups of participants with higher vulnerability to psychological distress, depression, anxiety, insomnia, suicidal ideation, and trauma were identified from the mixed models. Predicted probabilities of elevated psychological distress, depression, anxiety, insomnia, and suicidal ideation were significantly higher for participants who reported exposure to one or more adverse childhood experiences (versus none) and who reported a mental illness history (versus none). Predicted probabilities of elevated psychological distress, depression, anxiety, insomnia, and suicidal ideation were also higher for participants who identified as female (versus male), as another gender (versus male), as sexuality diverse (versus heterosexual), and as unsure of their sexuality (versus sexuality diverse), *ps* < 0.007. Participants who identified as another gender also had higher predicted probabilities of psychological distress, depression, insomnia, and suicidal ideation compared to females, *ps* < 0.007. Predicted probabilities of psychological distress, depression, anxiety, and insomnia were significantly higher for participants who reported low perceived household wealth (versus high), *ps* < 0.007. Overall, a similar pattern of results was found for elevated symptoms of COVID-19 trauma but not bushfire trauma. For bushfire trauma, predicted probabilities of elevated symptoms were significant for female gender (versus males) and sexuality diversity (versus heterosexual), *ps* < 0.007. There were no significant comparisons for language spoken most at home, *ps* > 0.10.

Predicted probabilities of elevated symptoms of COVID-19 trauma were significantly higher for participants who reported COVID-19 diagnosis/quarantine (versus did not), *ps* < 0.007. Predicted probabilities of elevated insomnia, suicidal ideation and bushfire trauma were significantly higher for participants who reported exposure to bushfire personal harm (versus no exposure), *p*s < 0.004. COVID-19 diagnosis/quarantine and bushfire personal harm did not significantly predict the remaining mental health variables, *ps* > 0.01, but probabilities were trending in the expected direction.

### Secondary analyses

Likelihood ratio tests demonstrated that the model including two-way interactions between disaster (COVID-19 diagnosis/quarantine, bushfire personal harm) and adverse childhood experiences was a better fit than the main effect model for depression, *χ*^2^(1) = 6.06, *p* = 0.048. A similar result was found for the model including two-way interactions between disaster and mental illness history for suicidal ideation, *χ*^2^(2) = 6.86, *p* = 0.03, and bushfire trauma, *χ*^2^(2) = 9.12, *p* = 0.01. No other two-way interactions were significant (see Additional file 1: Table S13–S15 in the Supplementary Materials for likelihood ratio tests, predicted probabilities, and simple effects analyses).

Adverse childhood experiences were associated with higher odds of depression than no adverse childhood experiences, but the effect was larger for participants who experienced COVID-19 diagnosis/quarantine (OR = 11.07, CI 95% [3.81, 32.10]) compared to those who did not (OR = 5.89, CI 95% [2.77, 12.50]). Similarly, the effect was larger for participants who reported being exposed to bushfire personal harm (OR = 14.86, CI 95% [3.28, 67.37]) compared to those who did not (OR = 4.38, CI 95% [2.86, 6.73]). Participants with mental illness history had higher odds of suicidal ideation and bushfire trauma compared to participants with no mental illness history. For suicidal ideation, the effect of mental illness history was only significant for participants who did not experience COVID-19 diagnosis/quarantine (OR = 2.68, CI 95% [1.67, 4.29]) and bushfire personal harm (OR = 2.60, CI 95% [1.55, 4.33]). For bushfire trauma, the effect of mental illness history was significant for participants who experienced COVID-19 diagnosis/quarantine (OR = 4.10, CI 95% [1.82, 9.22]) and for participants who did not experience bushfire personal harm (OR = 3.07, CI 95% [1.63, 5.79]).

## Discussion

The primary aim of the current study was to explore the effects of COVID-19 diagnosis/quarantine and bushfire personal harm on the mental health of Australian adolescents between 2020–2021 while controlling for personal risk factors. The secondary aim was to identify whether theoretically informed personal risk factors moderated vulnerability to these adverse environmental events.

COVID-19 diagnosis/quarantine and bushfire personal harm were differentially associated elevated mental health symptoms in adolescents. Controlling for personal risk factors and bushfire personal harm, the probability of elevated COVID-19 trauma was 9% higher for adolescents who reported a COVID-19 diagnosis and/or quarantine than those who did not (i.e., 31% vs 22%). Controlling for personal risk factors and COVID-19 diagnosis/quarantine, the probabilities of elevated insomnia, suicidal ideation and bushfire trauma were 9%, 4%, and 19% higher, respectively, for adolescents who reported bushfire personal harm than those who did not (i.e., 25% vs 17%, 9% vs 5%, and 44% vs 25%). This pattern of results is similar to those found in representative samples of Australian adults [12, 13]. In these studies, exposure to COVID-19 itself did not harm mental health in adults; COVID-19 related financial distress and social impairment were associated with higher symptom levels of anxiety and depression in the first three months of the pandemic [12, 13]. The results of the current study might be attributable to the way that we operationalised COVID-19 exposure. Focusing on COVID-19 diagnosis and quarantine likely does not capture variability in adolescents’ experiences (e.g., differences between local areas, family factors, social environments), or capture other core features of COVID-19 including uncertainty about the future and risk of death/severe illness. Overall, the type, degree, and specific characteristics of disasters experienced by adolescents are important to consider when evaluating effects on mental health.

Contrary to the theory of syndemics, we did not find evidence for interaction effects between COVID-19 diagnosis/quarantine and bushfire personal harm on elevated mental health symptoms in adolescents. Lack of support for interactive effects in the current study provides support for the idea that it is the type or severity of disasters, rather than the number, that is particularly important for mental health outcomes [10]. That is, because the experiences of COVID-19 and Black Summer bushfires in our adolescent cohort were qualitatively different (e.g., proximity, length of exposure, physical exposure, degree of social isolation), they resulted in separate or different stress reactivities. Quantifying different aspects of disasters across the community and individual level (e.g., mandated social isolation, perceived level of support, perceived risk of death), as well as family, financial, and environmental factors might tap into different synergistic effects. Another explanation for lack of interaction effects is insufficient power to detect significant differences between exposure groups. In particular, our criteria resulted in relatively small numbers of adolescents endorsing exposure to both COVID-19 diagnosis/quarantine and bushfire personal harm. It is important to note that the Future Proofing dataset was not specifically powered to explore complex interaction relationships in the sample at baseline.

The results from the current study demonstrated that some groups of adolescents had a higher probability of elevated mental health symptoms over and above self-reported COVID-19 diagnosis/quarantine and bushfire personal harm. Consistent with prior literature [e.g., 16, 17], these adolescents included those who reported adverse childhood experiences and a mental illness history, who identified as female, another gender (other than male), or sexuality diverse, and who were from low socio-economic families. The estimated effect sizes were generally larger than those for personal exposure to COVID-19 diagnosis/quarantine and bushfire personal harm, indicating the relative importance of individual vulnerability factors. Our exploratory interaction analyses also provided evidence that some adolescents respond differently to COVID-19 diagnosis/quarantine and bushfire personal harm. Relationships between disaster variables and personal risk factors (adverse childhood experiences, mental illness history) were generally sub-additive or additive. For example, exposure to both adverse childhood experiences and COVID-19 diagnosis/quarantine was associated with a 47% probability of elevated depression (i.e., 7% [COVID-19 diagnosis/quarantine without adverse childhood experiences] + 40% [adverse childhood experiences without COVID-19 diagnosis/quarantine] = 47%). In this case, adverse childhood experiences seem to account for most of the variance in depression compared to disaster exposure. These results indicate that addressing the complex social and psychological factors associated with mental ill health may be relevant in public health campaigns and disaster-response plans irrespective of the specific type of disaster. Further research is necessary to explore such interaction effects.

## Limitations and future research

The current study design is limited in that effects of COVID-19 and bushfires were only assessed at one point in time. There is a pressing need for prospective longitudinal designs, with measures of mental health before and after disasters, to examine changes in the same individuals over time. Once data collection has been completed (anticipated in 2026), the Future Proofing Study dataset will provide opportunities to explore mental health patterns and trajectories in Australian adolescents following large-scale community disasters including COVID-19, bushfires, and floods.

Data collection did not overlap with the 2019–2020 bushfires; the earliest data collection began in August 2020, approximately seven months after the worst of the bushfires (particularly in the New South Wales/Victoria regions). This sampling timeframe may have diluted bushfire effects due to the relative proximity and ongoing nature of COVID-19. Given that other research has shown that the first year following a disaster is the time of peak symptoms [4], our results likely provide a conservative estimate of the mental health impacts of bushfires. Understanding of youth mental health responses to disasters may be advanced by exploring objective indicators of disaster exposure.

## Conclusions

Different effects of COVID-19 and the bushfires on adolescent mental health were observed, and there were no interactive effects between these disasters. Future longitudinal research with objective indicators of exposure variables is needed to understand the long-term effects of disasters on adolescent mental health in Australia. A national disaster response plan focusing on complex social and psychological factors may prevent and mitigate mental health harms in adolescents.

## Supplementary Information

**Additional file 1: Table S1.** Likelihood ratio tests for each sample characteristic, which compare mixed models with and without cohort. **Table S2.** Descriptive comparison of sample characteristics by cohort (N=5866). **Table S3.** Likelihood ratio tests for each sample characteristic, which compare mixed models with and without state. **Table S4.** Descriptive comparison of sample characteristics by state (N=5866). **Table S5.** Proportion of participants scoring within the normal and elevated ranges for each mental health measure. **Table S6.** Binomial models predicting elevated psychological distress from personal risk factors, COVID-19 diagnosis/quarantine, bushfire personal harm, and COVID-19 diagnosis/quarantine × bushfire personal harm. Main effects and interactions between disaster variables were added sequentially in Models 2, 3 and 4. Model 5 is the fixed effects model of the best fitting model (Model 2). **Table S7.** Binomial models predicting elevated depression from personal risk factors, COVID-19 diagnosis/quarantine, bushfire personal harm, and COVID-19 diagnosis/quarantine × bushfire personal harm. Main effects and interactions between disaster variables were added sequentially in Models 2, 3 and 4. Model 5 is the fixed effects model of the best fitting model (Model 3). **Table S8.** Binomial models predicting elevated anxiety from personal risk factors, COVID-19 diagnosis/quarantine, bushfire personal harm, and COVID-19 diagnosis/quarantine × bushfire personal harm. Main effects and interactions between disaster variables were added sequentially in Models 2, 3 and 4. Model 5 is the fixed effects model of the best fitting model (Model 3). **Table S9.** Binomial models predicting elevated insomnia from personal risk factors, COVID-19 diagnosis/quarantine, bushfire personal harm, and COVID-19 diagnosis/quarantine × bushfire personal harm. Main effects and interactions between disaster variables were added sequentially in Models 2, 3 and 4. Model 5 is the fixed effects model of the best fitting model (Model 3). **Table S10.** Binomial models predicting elevated suicidal ideation from personal risk factors, COVID-19 diagnosis/quarantine, bushfire personal harm, and COVID-19 diagnosis/quarantine × bushfire personal harm. Main effects and interactions between disaster variables were added sequentially in Models 2, 3 and 4. Model 5 is the fixed effects model of the best fitting model (Model 3). **Table S11.** Binomial models predicting elevated COVID-19 trauma from personal risk factors, COVID-19 diagnosis/quarantine, bushfire personal harm, and COVID-19 diagnosis/quarantine × bushfire personal harm. Main effects and interactions between disaster variables were added sequentially in Models 2, 3 and 4. Model 5 is the fixed effects model of the best fitting model (Model 2). **Table S12.** Binomial models predicting elevated bushfire trauma from personal risk factors, COVID-19 diagnosis/quarantine, bushfire personal harm, and COVID-19 diagnosis/quarantine × bushfire personal harm. Main effects and interactions between disaster variables were added sequentially in Models 2, 3 and 4. Model 5 is the fixed effects model of the best fitting model (Model 3). **Table S13.** Likelihood ratio tests, which compare models with and without two-way interactions between personal risk factors (adverse childhood experiences, mental illness history, gender, sexual orientation, perceived household wealth) and disaster (COVID-19 diagnosis/quarantine, bushfire personal harm) for each mental health measure. Comparisons provide a direct test of the stress-diathesis model. **Table S14.** Predicted probabilities (standard error) for each level of the significant disaster × adverse childhood experiences and disaster × mental illness history interactions presented in Table S1 for depression, suicidal ideation, and bushfire trauma. **Table S15.** Simple effect comparisons for the significant disaster × adverse childhood experiences and disaster × mental illness history interactions for presented in Table S1 for depression, suicidal ideation, and bushfire trauma.

## Data Availability

The dataset used and analysed during the current study is available from the corresponding author on reasonable request.
