# Supplementary material for: The impact of COVID-19 and bushfires on the mental health of Australian adolescents: a cross-sectional study
Source: Child Adolesc Psychiatry Ment Health. 2023 Mar 9;17:34. doi: 10.1186/s13034-023-00583-1 (PMC9998012; doi:10.1186/s13034-023-00583-1)
Supplement: Supplementary file 1 — Additional file 1: Table S1. Likelihood ratio tests for each sample characteristic, which compare mixed models with and without cohort. Table S2. Descriptive comparison of sample characteristics by cohort (N=5866). Table S3. Likelihood ratio tests for each sample characteristic, which compare mixed models with and without state. Table S4. Descriptive comparison of sample characteristics by state (N=5866). Table S5. Proportion of participants scoring within the normal and elevated ranges for each mental health measure. Table S6. Binomial models predicting elevated psychological distress from personal risk factors, COVID-19 diagnosis/quarantine, bushfire personal harm, and COVID-19 diagnosis/quarantine × bushfire personal harm. Main effects and interactions between disaster variables were added sequentially in Models 2, 3 and 4. Model 5 is the fixed effects model of the best fitting model (Model 2). Table S7. Binomial models predicting elevated depression from personal risk factors, COVID-19 diagnosis/quarantine, bushfire personal harm, and COVID-19 diagnosis/quarantine × bushfire personal harm. Main effects and interactions between disaster variables were added sequentially in Models 2, 3 and 4. Model 5 is the fixed effects model of the best fitting model (Model 3). Table S8. Binomial models predicting elevated anxiety from personal risk factors, COVID-19 diagnosis/quarantine, bushfire personal harm, and COVID-19 diagnosis/quarantine × bushfire personal harm. Main effects and interactions between disaster variables were added sequentially in Models 2, 3 and 4. Model 5 is the fixed effects model of the best fitting model (Model 3). Table S9. Binomial models predicting elevated insomnia from personal risk factors, COVID-19 diagnosis/quarantine, bushfire personal harm, and COVID-19 diagnosis/quarantine × bushfire personal harm. Main effects and interactions between disaster variables were added sequentially in Models 2, 3 and 4. Model 5 is the fixed effects model of the [file 13034_2023_583_MOESM1_ESM.docx]

**Supplementary Materials**

**Detailed Descriptive Statistics**

***Bushfire Exposure and Impact***

Of the young people that did report living or going to school in an area that came under threat (*n=*899, 15.5%), 299 (33.3%) reported being evacuated from home or school, 265 (29.5%) reported that buildings in their home suburb were damaged or destroyed, 44 (4.9%) reported that home or possessions were damaged or destroyed, 268 (29.8%) reported that a friend or family member’s home or possessions were damaged or destroyed, 120 (13.3%) reported suffering injuries, 144 (16%) reported that a friend or relative suffered injuries, and 83 (9.2%) reported owning a pet/animal that suffered during the fires. Of these young people, most reported feeling “a bit” (*n=*514, 57.2%) or “very” (*n=*192, 21.4%) frightened or upset. Less than one-quarter (*n=*193, 21.5%) did not report feeling frightened or upset at all.

**Supplementary Tables**

**Table S1**

*Likelihood ratio tests for each sample characteristic, which compare mixed models with and without cohort*

| Sample Characteristics | Likelihood Ratio Test |
| --- | --- |
| Age | *χ2*(1)=4.22, *p*=.04^*^ |
| Adverse childhood experiences | *χ2*(1)=0.27, *p*=.61 |
| Mental illness history | *χ2*(1)=1.33, *p*=.25 |
| Gender identification | *χ2*(1)=0.38, *p*=.54 |
| Sexual orientation | *χ2*(1)=1.94, *p*=.16 |
| Perceived household wealth | *χ2*(1)=3.96, *p*=.05 |
| Language spoken most at home | *χ2*(1)=0.28, *p*=.60 |
| COVID-19 diagnosis/quarantine | *χ2*(1)=6.90, *p*=.009^*^ |
| Bushfire personal harm | *χ2*(1)=0.50, *p*=.48 |
| Psychological distress | *χ2*(1)=1.46, *p*=.23 |
| Depression | *χ2*(1)=0.24, *p*=.62 |
| Anxiety | *χ2*(1)=0.54, *p*=.46 |
| Insomnia | *χ2*(1)=0.55, *p*=.46 |
| Suicidal ideation | *χ2*(1)=0.20, *p*=.66 |
| COVID-19 trauma | *χ2*(1)=1.52, *p*=.22 |
| Bushfire trauma | *χ2*(1)=0.30, *p*=.56 |

Comparisons for each characteristic were made to the baseline model that included only the random effect of school. The best-fitting models for age and COVID-19 diagnosis/quarantine included cohort as a fixed effect. Despite these statistically significant differences, cohorts are conceptually similar (see Table S2). ^*^*p*<.05.

**Table S2**

*Descriptive comparison of sample characteristics by cohort (N=5866)*

| **Sample Characteristics** | **2020** | | **2021** |
| --- | --- | --- | --- |
|  | n=1911 | | n=3955 |
| **Mean age (SD)** | 14.04 (0.42) | | 13.84 (0.54) |
| **Adverse childhood experiences (n, %)** | | | |
| No | 601 (31.4%) | | 1226 (31) |
| Yes | 1310 (68.6) | | 2718 (68.7) |
| Prefer not to say | 0 (0) | | 11 (0.3) |
| **Mental illness history (n, %)** | | | |
| No | 1548 (81) | | 3272 (82.7) |
| Yes | 363 (19) | | 683 (17.3) |
| **Gender identification (n, %)** | | | |
| Male | 910 (47.6) | | 1752 (44.3) |
| Female | 936 (49) | | 1989 (50.3) |
| Other | 30 (1.6) | | 86 (2.2) |
| Prefer not to say | 11 (0.6) | | 54 (1.4) |
| **Sexual orientation (n, %)** | | | |
| Heterosexual or straight | 1370 (74.9) | | 2731 (71.6) |
| Sexuality diverse | 190 (10.4) | | 538 (14.1) |
| Unsure | 173 (9.5) | | 331 (8.7) |
| Prefer not to say | 83 (4.5) | | 197 (5.2) |
| **Perceived household wealth (n, %)** | | | |
| High | 808 (42.3) | | 1788 (45.2) |
| Low | 817 (42.8) | | 1622 (41) |
| Prefer not to say | 286 (15) | | 545 (13.8) |
| **Language spoken most at home (n, %)** | | | |
| English | | 1774 (92.8) | 3707 (93.8) |
| Other | | 137 (7.2) | 247 (6.2) |
| **Covid-19 diagnosis/quarantine (n, %)** | | | |
| No | 1701 (89) | | 3363 (85) |
| Yes | 210 (11) | | 592 (15) |
| **Bushfire personal harm (n, %)** | | | |
| No | 1757 (91.9) | | 3725 (94.2) |
| Yes | 154 (8.1) | | 230 (5.8) |
| **Psychological distress (n, %)** | | | |
| Normal range | 1340 (70.2) | | 2628 (66.5) |
| Elevated range | 568 (29.8) | | 1326 (33.5) |
| **Depression (n, %)** | | | |
| Normal range | 1621 (84.8) | | 3324 (84.1) |
| Elevated range | 290 (15.2) | | 630 (15.9) |
| **Anxiety (n, %)** | | | |
| Normal range | 1543 (80.9) | | 3194 (80.8) |
| Elevated range | 364 (19.1) | | 757 (19.2) |
| **Insomnia (n, %)** | | | |
| Normal range | 1686 (88.5) | | 3491 (88.6) |
| Elevated range | 220 (11.5) | | 451 (11.4) |
| **Suicidal ideation (n, %)** | | | |
| Normal range | 1662 (94.5) | | 3452 (95.1) |
| Elevated range | 97 (5.5) | | 177 (4.9) |
| **COVID-19 trauma (n, %)** | | | |
| Normal range | 1562 (83.3) | | 3140 (81.1) |
| Elevated range | 314 (16.7) | | 732 (18.9) |
| **Bushfire trauma (n, %)** | | | |
| Normal range | 303 (79.3) | | 400 (77.4) |
| Elevated range | 79 (20.7) | | 117 (22.6) |

**Table S3**

*Likelihood ratio tests for each sample characteristic, which compare mixed models with and without state*

| Sample Characteristics | Likelihood Ratio Test |
| --- | --- |
| Age | *χ2*(4)=14.80, *p*=.005^*^ |
| Adverse childhood experiences | *χ2*(4)=1.74, *p*=.78 |
| Mental illness history | *χ2*(4)=8.12, *p*=.09 |
| Gender identification | *χ2*(4)=11.33, *p*=.02^*^ |
| Sexual orientation | *χ2*(4)=1.50, *p*=.83 |
| Perceived household wealth | *χ2*(4)=7.40, *p*=.11 |
| Language spoken most at home | *χ2*(4)=2.67, *p*=.61 |
| COVID-19 diagnosis/quarantine | *χ2*(4)=1.80, *p*=.77 |
| Bushfire personal harm | *χ2*(4)=11.70, *p*=.02^*^ |
| Psychological distress | *χ2*(4)=5,62, *p*=.34 |
| Depression | *χ2*(4)=4.20, *p*=.62 |
| Anxiety | *χ2*(4)=2.40, *p*=.66 |
| Insomnia | *χ2*(4)=1.53, *p*=.82 |
| Suicidal ideation | *χ2*(4)=1.14, *p*=.89 |
| COVID-19 trauma | *χ2*(4)=14.34, *p*=.006^*^ |
| Bushfire trauma | *χ2*(4)=4.80, *p*=.31 |

Comparisons for each characteristic were made to the baseline model that included only the random effect of school. The best-fitting models for age, gender, bushfire personal harm, and COVID-19 trauma included state as a fixed effect. Despite these statistically significant differences, cohorts are conceptually similar (see Table S4). ^*^*p*<.05.

**Table S4**

*Descriptive comparison of sample characteristics by state (N=5866)*

| **Sample Characteristics** | **NSW** | **QLD** | **SA** | **VIC** | **WA** |
| --- | --- | --- | --- | --- | --- |
|  | n=4944 | n=196 | n=89 | n=454 | n=183 |
| **Mean age (SD)** | 13.90 (0.51) | 13.79 (0.54) | 13.90 (0.32) | 14.11 (0.55) | 13.65 (0.46) |
| **Adverse childhood experiences (n, %)** | | | | | |
| No | 1549 (31.3) | 54 (27.6) | 31 (34.8) | 140 (30.8) | 53 (29) |
| Yes | 3385 (68.5) | 141 (71.9) | 58 (65.2) | 314 (69.2) | 130 (71) |
| Prefer not to say | 10 (0.2) | 1 (0.5) | 0 (0) | 0 (0) | 0 (0) |
| **Mental illness history (n, %)** | | | | | |
| No | 4041 (81.7) | 156 (79.6) | 80 (89.9) | 395 (87) | 148 (80.9) |
| Yes | 903 (18.3) | 40 (20.4) | 9 (10.1) | 59 (13) | 35 (19.1) |
| **Gender identification (n, %)** | | | | | |
| Male | 2310 (46.7) | 78 (39.8) | 1 (1.1) | 196 (43.2) | 77 (42.1) |
| Female | 2404 (48.6) | 112 (57.1) | 78 (87.6) | 234 (51.1) | 97 (53) |
| Other | 152 (3.1) | 6 (3) | 5 (5.6) | 10 (2.2) | 8 (4.3) |
| Prefer not to say | 78 (1.1) | 0 (1.5) | 5 (2.2) | 14 (1.5) | 1 (0.5) |
| **Sexual orientation (n, %)** | | | | | |
| Heterosexual or straight | 3445 (72.6) | 149 (76.4) | 54 (60.7) | 330 (74.2) | 123 (72.4) |
| Sexuality diverse | 603 (12.7) | 23 (11.8) | 12 (13.5) | 66 (14.8) | 24 (14.1) |
| Unsure | 432 (9.1) | 14 (7.2) | 15 (16.9) | 29 (6.5) | 14 (8.2) |
| Prefer not to say | 240 (5.1) | 9 (4.6) | 7 (7.9) | 16 (3.6) | 8 (4.7) |
| **Perceived household wealth (n, %)** | | | | | |
| High | 2138 (43.2) | 100 (51) | 44 (49.4) | 243 (53.5) | 71 (38.8) |
| Low | 2083 (42.1) | 68 (34.7) | 36 (40.4) | 163 (35.9) | 89 (48.6) |
| Prefer not to say | 723 (14.6) | 28 (14.3) | 9 (10.1) | 48 (10.6) | 23 (12.6) |
| **Language spoken most at home (n, %)** | | | | | |
| English | 4626 (93.6) | 189 (96.4) | 69 (77.5) | 422 (93) | 175 (95.6) |
| Other | 317 (6.4) | 7 (3.6) | 20 (22.5) | 32 (7) | 8 (4.4) |
| **COVID-19 diagnosis/quarantine (n, %)** | | | | | |
| No | 4268 (86.3) | 173 (88.3) | 80 (89.9) | 389 (85.7) | 154 (84.2) |
| Yes | 676 (13.7) | 23 (11.7) | 9 (10.1) | 65 (14.3) | 29 (15.8) |
| **Bushfire personal harm (n, %)** | | | | | |
| No | 4584 (92.7) | 194 (99) | 87 (97.8) | 444 (97.8) | 173 (94.5) |
| Yes | 360 (7.3) | 2 (1) | 2 (2.2) | 10 (2.2) | 10 (5.5) |
| **Psychological distress (n, %)** | | | | | |
| Normal range | 3372 (68.3) | 141 (71.9) | 53 (59.6) | 283 (62.3) | 119 (65) |
| Elevated range | 1568 (31.7) | 55 (28.1) | 36 (40.4) | 171 (37.7) | 64 (35) |
| **Depression (n, %)** | | | | | |
| Normal range | 4163 (84.2) | 178 (91.3) | 72 (80.9) | 378 (83.3) | 154 (84.2) |
| Elevated range | 781 (15.8) | 17 (8.7) | 17 (19.1) | 76 (16.7) | 29 (15.8) |
| **Anxiety (n, %)** | | | | | |
| Normal range | 3994 (80.9) | 165 (84.2) | 69 (77.5) | 359 (79.1) | 150 (82.0) |
| Elevated range | 942 (19.1) | 31 (15.8) | 20 (22.5) | 95 (20.9) | 33 (18) |
| **Insomnia (n, %)** | | | | | |
| Normal range | 4354 (88.4) | 179 (91.3) | 78 (87.6) | 403 (88.8) | 163 (89.6) |
| Elevated range | 573 (11.6) | 17 (8.7) | 11 (12.4) | 51 (11.2) | 19 (10.4) |
| **Suicidal ideation (n, %)** | | | | | |
| Normal range | 4283 (94.8) | 186 (96.9) | 73 (93.6) | 409 (95.8) | 163 (95.3) |
| Elevated range | 237 (5.2) | 6 (3.1) | 5 (6.4) | 18 (4.2) | 8 (4.7) |
| C**OVID-19 trauma (n, %)** | | | | | |
| Normal range | 3979 (82.2) | 172 (87.8) | 73 (82) | 334 (74.6) | 144 (82.3) |
| Elevated range | 861 (17.8) | 24 (12.2) | 16 (18) | 114 (25.4) | 31 (17.7) |
| **Bushfire trauma (n, %)** | | | | | |
| Normal range | 658 (77.8) | 9 (100) | 3 (75) | 18 (81.8) | 15 (83.3) |
| Elevated range | 188 (22.2) | 0 (0) | 1 (25) | 4 (18.2) | 3 (16.7) |

*Note.* NSW=New South Wales; QLD=Queensland; SA=South Australia; VIC=Victoria; WA=Western Australia.

**Table S5**

*Proportion of participants scoring within the normal and elevated ranges for each mental health measure*

| **Mental Health Measures** | N (%) |
| --- | --- |
| **Psychological distress** (*M*=11.43, *SD*=5.10) | |
| Normal range | 3968 (67.7) |
| Elevated range | 1894 (32.3) |
| **Depression** (*M*=7.52, *SD*=6.35) | |
| Normal range | 4945 (84.3) |
| Elevated range | 920 (15.7) |
| **Anxiety** (*M*=8.35, *SD*=5.57) | |
| Normal range | 4737 (80.9) |
| Elevated range | 1121 (19.1) |
| **Insomnia** (*M*=7.17, *SD*=5.76) | |
| Normal range | 5177 (88.5) |
| Elevated range | 671 (11.5) |
| **Suicidal ideation** (*M*=2,92, *SD*=7.65) | |
| Normal range | 5114 (94.9) |
| Elevated range | 274 (5.1) |
| **Covid-19 trauma** (*M*=2.46, *SD*=2.17) | |
| Normal range | 4702 (81.8) |
| Elevated range | 1046 (18.2) |
| **Bushfire trauma** (*M*=2.64, *SD*=2.57) | |
| Normal range | 703 (78.2) |
| Elevated range | 196 (21.8) |

**Table S6**

*Binomial models predicting elevated psychological distress from personal risk factors, COVID-19 diagnosis/quarantine, bushfire personal harm, and COVID-19 diagnosis/quarantine × bushfire personal harm. Main effects and interactions between disaster variables were added sequentially in Models 2, 3 and 4. Model 5 is the fixed effects model of the best fitting model (Model 2)*

| Explanatory Variables | Mixed Effects Models | | | | Fixed Effects Model |
| --- | --- | --- | --- | --- | --- |
|  | Model 1 | Model 2 | Model 3 | Model 4 | Model 5 |
| Fixed effects, OR (95% CI) | | | | | |
| Adverse childhood experiences (ref=no) | 3.24 (2.71, 3.76)^*^ | 3.21 (2.69, 3.73)^*^ | 3.21 (2.69, 3.73)^*^ | 3.21 (2.69, 3.74)^*^ | 3.20 (3.04, 3.36)^*^ |
| Mental illness history (ref=no) | 3.40 (2.85, 3.95)^*^ | 3.38 (2.83, 3.93)^*^ | 3.39 (2.84, 3.94)^*^ | 3.38 (2.83, 3.93)^*^ | 3.37 (3.21, 3.53)^*^ |
| Gender (ref=male) | | | | | |
| Female | 3.66 (3.11, 4.20)^*^ | 3.65 (3.11, 4.19)^*^ | 3.65 (3.11, 4.19)^*^ | 3.65 (3.11, 4.19)^*^ | 3.61 (3.47, 3.75)^*^ |
| Other | 8.41 (4.68, 12.14)^*^ | 8.30 (4.62, 11.98)^*^ | 8.30 (4.62, 11.99)^*^ | 8.33 (4.63, 12.03)^*^ | 8.10 (7.66, 8.54)^*^ |
| Prefer not to say | 5.24 (2.60, 7.88)^*^ | 5.21 (2.58, 7.85)^*^ | 5.21 (2.58, 7.84)^*^ | 5.22 (2.58, 7.85)^*^ | 5.33 (4.83, 5.83)^*^ |
| Sexual orientation (ref=heterosexual) | | | | | |
| Sexuality diverse | 3.29 (2.62, 3.96)^*^ | 3.30 (2.63, 3.97)^*^ | 3.30 (2.63, 3.97)^*^ | 3.30 (2.63, 3.97)^*^ | 3.34 (3.14, 3.54)^*^ |
| Unsure | 1.58 (1.24, 1.93)^*^ | 1.58 (1.24, 1.92)^*^ | 1.58 (1.24, 1.92)^*^ | 1.58 (1.24, 1.92)^*^ | 1.58 (1.37, 1.80)^*^ |
| Prefer not to say | 1.47 (1.04, 1.90) | 1.46 (1.03, 1.89) | 1.46 (1.03, 1.89) | 1.46 (1.03, 1.89) | 1.46 (1.17, 1.75) |
| Perceived household wealth (ref=high) | | | | | |
| Low | 1.42 (1.22, 1.62)^*^ | 1.41 (1.21, 1.61)^*^ | 1.41 (1.22, 1.61)^*^ | 1.41 (1.21, 1.61)^*^ | 1.42 (1.28, 1.56)^*^ |
| Prefer not to say | 1.17 (0.93, 1.41) | 1.18 (0.94, 1.41) | 1.18 (0.94, 1.41) | 1.18 (0.94, 1.41) | 1.17 (0.97, 1.37) |
| Language spoken most at home (ref=English) | 1.19 (0.87, 1.51) | 1.18 (0.87, 1.50) | 1.18 (0.87, 1.50) | 1.18 (0.87, 1.50) | 1.20 (0.94, 1.46) |
| COVID-19 diagnosis/quarantine (ref=no) |  | 1.25 (1.02, 1.48) | 1.25 (1.02, 1.48) | 1.27 (1.03, 1.52) | 1.27 (1.08, 1.45) |
| Bushfire personal harm (ref=no) |  |  | 0.97 (0.71, 1.22) | 1.01 (0.71, 1.31) |  |
| COVID-19 diagnosis/quarantine × bushfire personal harm |  |  |  | 0.82 (0.32, 1.32) |  |
| Intercept | 0.05 (0.04, 0.05)^*^ | 0.04 (0.03, 0.05)^*^ | 0.04 (0.03, 0.05)^*^ | 0.04 (0.03, 0.05)^*^ | 0.04 (-0.16, 0.24)^*^ |
| Random Effects |  |  |  |  |  |
| *N* School | 122 | 122 | 122 | 122 |  |
| σ^2^ | 3.29 | 3.29 | 3.29 | 3.29 |  |
| *SD* | 0.22 | 0.21 | 0.21 | 0.21 |  |
| ICC (95% CI) | 0.05 (0.001, 0.08) | 0.04 (0.009, 0.08) | 0.04 (0.007, 0.09) | 0.04 (0.009, 0.9) |  |
| Model Statistics |  |  |  |  |  |
| Observations | 5601 | 5601 | 5601 | 5601 | 5601 |
| Log Likelihood | -2,807.92 | -2,805.18 | -2,805.14 | -2,804.94 | -2,808.49 |
| AIC | 5,641.84 | 5,638.35 | 5,640.29 | 5,641.89 | 5,642.98 |
| Marginal R^2^/Conditional R^2^ | .33/.34 | .33/.34 | .33/.34 | .33/.34 |  |
| LRT |  | *χ2*(1)=5.48, *p*=.02 | *χ2*(1)=0.06, *p*=.80 | *χ2*(1)=0.40, *p*=.53 | *χ2*(1)=6.62, *p*=.01 |

*Note:* OR=odds ratio (conditional on fixed effects); 95% CI=95% confidence intervals; ref=reference category, ICC=intraclass correlation coefficient (95% CI estimated via bootstrapped simulation); AIC=akaike information criterion; LRT=likelihood ratio test (Model 2 vs Model 3; Model 3 vs Model 4; Model 4 vs Model 5; Model 5 vs Model 2). Model 1=personal risk factors (fixed effects) + school (random intercepts); Model 2=personal risk factors + COVID-19 diagnosis/quarantine (fixed effects) + school (random intercepts); Model 3=personal risk factors + COVID-19 diagnosis/quarantine + bushfire personal harm (fixed effects) + school (random intercepts); Model 4=personal risk factors + COVID-19 diagnosis/quarantine + bushfire personal harm + COVID-19 diagnosis/quarantine × bushfire personal harm (fixed effects) + school (random intercepts); Model 5=personal risk factors + COVID-19 diagnosis/quarantine (fixed effects). Model 2 identified as best fitting model. ^*^*p*<.007.

**Table S7**

*Binomial models predicting elevated depression from personal risk factors, COVID-19 diagnosis/quarantine, bushfire personal harm, and COVID-19 diagnosis/quarantine × bushfire personal harm. Main effects and interactions between disaster variables were added sequentially in Models 2, 3 and 4. Model 5 is the fixed effects model of the best fitting model (Model 3)*

| Explanatory Variables | Mixed Effects Models | | | | Fixed Effects Model |
| --- | --- | --- | --- | --- | --- |
|  | Model 1 | Model 2 | Model 3 | Model 4 | Model 5 |
| Fixed effects, OR (95% CI) | | | | | |
| Adverse childhood experiences (ref=no) | 3.69 (2.79, 4.59)^*^ | 3.65 (2.75, 4.54)^*^ | 3.62 (2.73, 4.51)^*^ | 3.62 (2.73, 4.51)^*^ | 3.59 (3.34, 3.83)^*^ |
| Mental illness history (ref=no) | 3.26 (2.68, 3.84)^*^ | 3.24 (2.66, 3.81)^*^ | 3.22 (2.65, 3.79)^*^ | 3.22 (2.65, 3.80)^*^ | 3.19 (3.01, 3.36)^*^ |
| Gender (ref=male) | | | | | |
| Female | 2.42 (1.95, 2.89)^*^ | 2.41 (1.94, 2.87)^*^ | 2.41 (1.94, 2.87)^*^ | 2.41 (1.94, 2.87)^*^ | 2.39 (2.21, 2.58)^*^ |
| Other | 7.11 (4.24, 9.98)^*^ | 7.00 (4.17, 9.82)^*^ | 6.97 (4.15, 9.78)^*^ | 6.96 (4.15, 9.78)^*^ | 6.77 (6.37, 7.17)^*^ |
| Prefer not to say | 2.12 (1.02, 3.23)^*^ | 2.10 (1.01, 3.19)^*^ | 2.11 (1.01, 3.21)^*^ | 2.11 (1.01, 3.21)^*^ | 2.10 (1.58, 2.61)^*^ |
| Sexual orientation (ref=heterosexual) | | | | | |
| Sexuality diverse | 3.75 (2.95, 4.56)^*^ | 3.75 (2.95, 4.56)^*^ | 3.77 (2.96, 4.57)^*^ | 3.77 (2.96, 4.57)^*^ | 3.79 (3.57, 4.00)^*^ |
| Unsure | 1.45 (1.05, 1.85) | 1.44 (1.04, 1.85) | 1.45 (1.04, 1.85) | 1.45 (1.04, 1.85) | 1.45 (1.17, 1.72) |
| Prefer not to say | 1.82 (1.18, 2.47)^*^ | 1.81 (1.17, 2.45)^*^ | 1.81 (1.17, 2.46)^*^ | 1.81 (1.17, 2.46)^*^ | 1.82 (1.47, 2.16)^*^ |
| Perceived household wealth (ref=high) | | | | | |
| Low | 1.47 (1.21, 1.73)^*^ | 1.47 (1.20, 1.73)^*^ | 1.46 (1.20, 1.72)^*^ | 1.46 (1.20, 1.72)^*^ | 1.47 (1.29, 1.65)^*^ |
| Prefer not to say | 1.45 (1.09, 1.81)^*^ | 1.46 (1.09, 1.83)^*^ | 1.45 (1.09, 1.82)^*^ | 1.45 (1.09, 1.82)^*^ | 1.45 (1.20, 1.70)^*^ |
| Language spoken most at home (ref=English) | 1.17 (0.78, 1.55) | 1.15 (0.77, 1.54) | 1.16 (0.77, 1.54) | 1.16 (0.77, 1.54) | 1.15 (0.83, 1.48) |
| COVID-19 diagnosis/quarantine (ref=no) |  | 1.27 (0.99, 1.55) | 1.25 (0.97, 1.52) | 1.24 (0.95, 1.53) | 1.27 (1.06, 1.49) |
| Bushfire personal harm (ref=no) |  |  | 1.40 (0.98, 1.82) | 1.38 (0.91, 1.86) | 1.42 (1.12, 1.71) |
| COVID-19 diagnosis/quarantine × bushfire personal harm |  |  |  | 1.04 (0.34, 1.75) |  |
| Intercept | 0.01 (0.01, 0.02)^*^ | 0.01 (0.01, 0.02)^*^ | 0.01 (0.01, 0.02)^*^ | 0.01 (0.01, 0.02)^*^ | 0.01 (-0.27, 0.30)^*^ |
| Random Effects |  |  |  |  |  |
| *N* School | 122 | 122 | 122 | 122 |  |
| σ^2^ | 3.29 | 3.29 | 3.29 | 3.29 |  |
| *SD* | 0.28 | 0.27 | 0.27 | 0.27 |  |
| ICC (95% CI) | 0.07 (0.03, 0.14) | 0.07 (0.03, 0.12) | 0.07 (0.008, 0.11) | 0.07 (0.007, 0.10) |  |
| Model Statistics |  |  |  |  |  |
| Observations | 5600 | 5600 | 5600 | 5600 | 5600 |
| Log Likelihood | -1,952.28 | -1,950.06 | -1,947.71 | -1,947.70 | -1,951.55 |
| AIC | 3,930.56 | 3,928.13 | 3,925.41 | 3,927.39 | 3,931.11 |
| Marginal R^2^/Conditional R^2^ | .31/.32 | .31/.32 | .31/.32 | .31/.32 |  |
| LRT |  | *χ2*(1)=4.43, *p*=.04 | *χ2*(1)=4.72, *p*=.03 | *χ2*(1)=0.02, *p*=.90 | *χ2*(1)=7.70, *p*=.006 |

*Note:* OR=odds ratio (conditional on fixed effects); 95% CI=95% confidence intervals; ref=reference category, ICC=intraclass correlation coefficient (95% CI estimated via bootstrapped simulation); AIC=akaike information criterion; LRT=likelihood ratio test (Model 2 vs Model 3; Model 3 vs Model 4; Model 4 vs Model 5; Model 5 vs Model 2). Model 1=personal risk factors (fixed effects) + school (random intercepts); Model 2=personal risk factors + COVID-19 diagnosis/quarantine (fixed effects) + school (random intercepts); Model 3=personal risk factors + COVID-19 diagnosis/quarantine + bushfire personal harm (fixed effects) + school (random intercepts); Model 4=personal risk factors + COVID-19 diagnosis/quarantine + bushfire personal harm + COVID-19 diagnosis/quarantine × bushfire personal harm (fixed effects) + school (random intercepts); Model 5=personal risk factors + COVID-19 diagnosis/quarantine (fixed effects). Model 3 identified as best fitting model. ^*^*p*<.007.

**Table S8**

*Binomial models predicting elevated anxiety from personal risk factors, COVID-19 diagnosis/quarantine, bushfire personal harm, and COVID-19 diagnosis/quarantine × bushfire personal harm. Main effects and interactions between disaster variables were added sequentially in Models 2, 3 and 4. Model 5 is the fixed effects model of the best fitting model (Model 3)*

| Explanatory Variables | Mixed Effects Models | | | | Fixed Effects Model |
| --- | --- | --- | --- | --- | --- |
|  | Model 1 | Model 2 | Model 3 | Model 4 | Model 5 |
| Fixed effects, OR (95% CI) | | | | | |
| Adverse childhood experiences (ref=no) | 2.42 (1.95, 2.89)^*^ | 2.40 (1.93, 2.87)^*^ | 2.38 (1.91, 2.85)^*^ | 2.38 (1.91, 2.85)^*^ | 0.85 (0.66, 1.05)^*^ |
| Mental illness history (ref=no) | 3.31 (2.75, 3.88)^*^ | 3.29 (2.73, 3.86)^*^ | 3.28 (2.71, 3.84)^*^ | 3.28 (2.71, 3.84)^*^ | 1.16 (0.99, 1.33)^*^ |
| Gender (ref=male) | | | | | |
| Female | 4.87 (3.93, 5.81)^*^ | 4.85 (3.91, 5.79)^*^ | 4.85 (3.91, 5.78)^*^ | 4.85 (3.91, 5.78)^*^ | 1.54 (1.36, 1.72)^*^ |
| Other | 8.51 (5.14, 11.87)^*^ | 8.38 (5.06, 11.70)^*^ | 8.33 (5.02, 11.63)^*^ | 8.34 (5.03, 11.65)^*^ | 2.07 (1.68, 2.46)^*^ |
| Prefer not to say | 4.37 (2.19, 6.56)^*^ | 4.33 (2.17, 6.50)^*^ | 4.35 (2.18, 6.53)^*^ | 4.36 (2.18, 6.53)^*^ | 1.47 (0.98, 1.96)^*^ |
| Sexual orientation (ref=heterosexual) | | | | | |
| Sexuality diverse | 2.39 (1.89, 2.90)^*^ | 2.39 (1.89, 2.90)^*^ | 2.40 (1.90, 2.91)^*^ | 2.40 (1.90, 2.91)^*^ | 0.88 (0.68, 1.09)^*^ |
| Unsure | 1.31 (0.98, 1.64) | 1.30 (0.97, 1.63) | 1.30 (0.97, 1.63) | 1.30 (0.97, 1.63) | 0.27 (0.02, 0.52) |
| Prefer not to say | 1.59 (1.06, 2.13)^*^ | 1.58 (1.06, 2.11)^*^ | 1.59 (1.06, 2.12) | 1.59 (1.06, 2.12) | 0.48 (0.15, 0.81)^*^ |
| Perceived household wealth (ref=high) | | | | | |
| Low | 1.47 (1.23, 1.71)^*^ | 1.47 (1.23, 1.71)^*^ | 1.46 (1.22, 1.70) | 1.46 (1.22, 1.70) | 0.39 (0.23, 0.56)^*^ |
| Prefer not to say | 1.27 (0.97, 1.56) | 1.27 (0.97, 1.57) | 1.27 (0.97, 1.57) | 1.27 (0.97, 1.57) | 0.24 (0.02, 0.47) |
| Language spoken most at home (ref=English) | 0.76 (0.51, 1.01) | 0.75 (0.50, 1.00) | 0.76 (0.51, 1.01) | 0.76 (0.51, 1.01) | -0.25 (-0.58, 0.07) |
| COVID-19 diagnosis/quarantine (ref=no) |  | 1.24 (0.98, 1.49) | 1.22 (0.97, 1.47) | 1.22 (0.96, 1.49) | 0.22 (0.02, 0.43) ^*^ |
| Bushfire personal harm (ref=no) |  |  | 1.45 (1.04, 1.86) | 1.47 (0.99, 1.94) | 0.39 (0.12, 0.66) |
| COVID-19 diagnosis/quarantine × bushfire personal harm |  |  |  | 0.95 (0.34, 1.56) |  |
| Intercept | 0.02 (0.02, 0.03) | 0.02 (0.02, 0.03) | 0.02 (0.02, 0.03) | 0.02 (0.02, 0.03) | -3.89 (-4.14, -3.63) |
| Random Effects |  |  |  |  |  |
| *N* School | 122 | 122 | 122 | 122 |  |
| σ^2^ | 3.29 | 3.29 | 3.29 | 3.29 |  |
| *SD* | 0.37 | 0.36 | 0.35 | 0.35 |  |
| ICC (95% CI) | 0.12 (0.07, 0.18) | 0.11 (0.05, 0.17) | 0.11 (0.05, 0.16) | 0.11 (0.05, 0.17) |  |
| Model Statistics |  |  |  |  |  |
| Observations | 5601 | 5601 | 5601 | 5601 | 5601 |
| Log Likelihood | -2,234.95 | -2,232.92 | -2,229.72 | -2,229.71 | -2,240.28 |
| AIC | 4,495.90 | 4,493.84 | 4,489.44 | 4,491.41 | 4,508.56 |
| Marginal R^2^/Conditional R^2^ | .31/.33 | .31/.33 | .31/.33 | .31/.33 |  |
| LRT |  | *χ2*(1)=4.06, *p*=.04 | *χ2*(1)=6.40, *p*=.01 | *χ2*(1)=0.03, *p*=.87 | *χ2*(1)=21,11, *p*<.001 |

*Note:* OR=odds ratio (conditional on fixed effects); 95% CI=95% confidence intervals; ref=reference category, ICC=intraclass correlation coefficient (95% CI estimated via bootstrapped simulation); AIC=akaike information criterion; LRT=likelihood ratio test (Model 2 vs Model 3; Model 3 vs Model 4; Model 4 vs Model 5; Model 5 vs Model 2). Model 1=personal risk factors (fixed effects) + school (random intercepts); Model 2=personal risk factors + COVID-19 diagnosis/quarantine (fixed effects) + school (random intercepts); Model 3=personal risk factors + COVID-19 diagnosis/quarantine + bushfire personal harm (fixed effects) + school (random intercepts); Model 4=personal risk factors + COVID-19 diagnosis/quarantine + bushfire personal harm + COVID-19 diagnosis/quarantine × bushfire personal harm (fixed effects) + school (random intercepts); Model 5=personal risk factors + COVID-19 diagnosis/quarantine (fixed effects). Model 3 identified as best fitting model. ^*^*p*<.007.

**Table S9**

*Binomial models predicting elevated insomnia from personal risk factors, COVID-19 diagnosis/quarantine, bushfire personal harm, and COVID-19 diagnosis/quarantine × bushfire personal harm. Main effects and interactions between disaster variables were added sequentially in Models 2, 3 and 4. Model 5 is the fixed effects model of the best fitting model (Model 3)*

| Explanatory Variables | Mixed Effects Models | | | | Fixed Effects Model |
| --- | --- | --- | --- | --- | --- |
|  | Model 1 | Model 2 | Model 3 | Model 4 | Model 5 |
| Fixed effects, OR (95% CI) | | | | | |
| Adverse childhood experiences (ref=no) | 2.06 (1.57, 2.55)^*^ | 2.03 (1.55, 2.52)^*^ | 2.01 (1.53, 2.50)^*^ | 2.01 (1.53, 2.49)^*^ | 2.03 (1.79, 2.27)^*^ |
| Mental illness history (ref=no) | 2.51 (2.03, 3.00)^*^ | 2.49 (2.01, 2.97)^*^ | 2.47 (2.00, 2.95)^*^ | 2.48 (2.00, 2.96)^*^ | 2.46 (2.27, 2.65)^*^ |
| Gender (ref=male) | | | | | |
| Female | 2.38 (1.86, 2.89)^*^ | 2.36 (1.85, 2.87)^*^ | 2.36 (1.85, 2.87)^*^ | 2.35 (1.84, 2.86)^*^ | 2.27 (2.06, 2.47)^*^ |
| Other | 5.12 (3.03, 7.22)^*^ | 5.02 (2.96, 7.08)^*^ | 4.97 (2.93, 7.00)^*^ | 4.92 (2.90, 6.95)^*^ | 4.62 (4.22, 5.02)^*^ |
| Prefer not to say | 3.22 (1.47, 4.96)^*^ | 3.18 (1.45, 4.90)^*^ | 3.19 (1.46, 4.91)^*^ | 3.17 (1.45, 4.89)^*^ | 3.03 (2.50, 3.56)^*^ |
| Sexual orientation (ref=heterosexual) | | | | | |
| Sexuality diverse | 2.33 (1.77, 2.88)^*^ | 2.32 (1.77, 2.88)^*^ | 2.33 (1.78, 2.89)^*^ | 2.34 (1.78, 2.90)^*^ | 2.29 (2.05, 2.52)^*^ |
| Unsure | 1.18 (0.81, 1.54) | 1.17 (0.80, 1.54) | 1.17 (0.80, 1.54) | 1.18 (0.81, 1.55) | 1.16 (0.85, 1.47) |
| Prefer not to say | 1.57 (0.96, 2.18) | 1.55 (0.95, 2.16) | 1.56 (0.95, 2.16) | 1.55 (0.94, 2.15) | 1.52 (1.13, 1.90) |
| Perceived household wealth (ref=high) | | | | | |
| Low | 1.48 (1.19, 1.76)^*^ | 1.48 (1.19, 1.76)^*^ | 1.47 (1.18, 1.75)^*^ | 1.47 (1.19, 1.75)^*^ | 1.47 (1.28, 1.66)^*^ |
| Prefer not to say | 1.21 (0.87, 1.54) | 1.21 (0.88, 1.55) | 1.21 (0.88, 1.55) | 1.22 (0.88, 1.56) | 1.23 (0.96, 1.50) |
| Language spoken most at home (ref=English) | 0.96 (0.60, 1.33) | 0.95 (0.59, 1.31) | 0.95 (0.59, 1.31) | 0.95 (0.59, 1.31) | 0.92 (0.55, 1.29) |
| COVID-19 diagnosis/quarantine (ref=no) |  | 1.27 (0.97, 1.56) | 1.24 (0.95, 1.53) | 1.17 (0.87, 1.47) | 1.26 (1.03, 1.49) |
| Bushfire personal harm (ref=no) |  |  | 1.60 (1.11, 2.10)^*^ | 1.44 (0.91, 1.96) | 1.63 (1.33, 1.92)^*^ |
| COVID-19 diagnosis/quarantine × bushfire personal harm |  |  |  | 1.51 (0.48, 2.53) |  |
| Intercept | 0.02 (0.01, 0.03)^*^ | 0.02 (0.01, 0.03)^*^ | 0.02 (0.01, 0.03)^*^ | 0.02 (0.01, 0.03)^*^ | 0.02 (-0.26, 0.30)^*^ |
| Random Effects |  |  |  |  |  |
| *N* School | 122 | 122 | 122 | 122 |  |
| σ^2^ | 3.29 | 3.29 | 3.29 | 3.29 |  |
| *SD* | 0.37 | 0.37 | 0.35 | 0.35 |  |
| ICC (95% CI) | 0.12 (0.07, 0.19) | 0.12 (0.03, 0.18) | 0.11 (0.05, 0.18) | 0.11 (0.05, 0.19) |  |
| Model Statistics |  |  |  |  |  |
| Observations | 5,601 | 5,601 | 5,601 | 5,601 | 5,601 |
| Log Likelihood | -1,754.69 | -1,752.77 | -1,748.54 | -1,747.84 | -1,754.39 |
| AIC | 3,535.37 | 3,533.54 | 3,527.08 | 3,527.68 | 3,536.78 |
| Marginal R^2^/Conditional R^2^ | .19/.22 | .19/.22 | .20/.22 | .20/.22 |  |
| LRT |  | *χ2*(1)=3.84, *p*=.05 | *χ2*(1)=8.46, *p*=.004 | *χ2*(1)=1.40, *p*=.24 | *χ2*(1)=11.70, *p<*.001 |

*Note:* OR=odds ratio (conditional on fixed effects); 95% CI=95% confidence intervals; ref=reference category, ICC=intraclass correlation coefficient; AIC=akaike information criterion; LRT=likelihood ratio test (Model 2 vs Model 3; Model 3 vs Model 4; Model 4 vs Model 5; Model 5 vs Model 2). Model 1=personal risk factors (fixed effects) + school (random intercepts); Model 2=personal risk factors + COVID-19 diagnosis/quarantine (fixed effects) + school (random intercepts); Model 3=personal risk factors + COVID-19 diagnosis/quarantine + bushfire personal harm (fixed effects) + school (random intercepts); Model 4=personal risk factors + COVID-19 diagnosis/quarantine + bushfire personal harm + COVID-19 diagnosis/quarantine × bushfire personal harm (fixed effects) + school (random intercepts); Model 5=personal risk factors + COVID-19 diagnosis/quarantine (fixed effects). Model 3 identified as best fitting model. ^*^*p*<.007.

**Table S10**

*Binomial models predicting elevated suicidal ideation from personal risk factors, COVID-19 diagnosis/quarantine, bushfire personal harm, and COVID-19 diagnosis/quarantine × bushfire personal harm. Main effects and interactions between disaster variables were added sequentially in Models 2, 3 and 4. Model 5 is the fixed effects model of the best fitting model (Model 3)*

| Explanatory Variables | Mixed Effects Models | | | | Fixed Effects Model |
| --- | --- | --- | --- | --- | --- |
|  | Model 1 | Model 2 | Model 3 | Model 4 | Model 5 |
| Fixed effects, OR (95% CI) | | | | | |
| Adverse childhood experiences (ref=no) | 6.42 (2.74, 10.09)^*^ | 6.34 (2.71, 9.98)^*^ | 6.27 (2.68, 9.86)^*^ | 6.27 (2.68, 9.86)^*^ | 6.19 (5.62, 6.76)^*^ |
| Mental illness history (ref=no) | 3.37 (2.44, 4.30)^*^ | 3.32 (2.40, 4.24)^*^ | 3.31 (2.39, 4.23)^*^ | 3.31 (2.39, 4.23)^*^ | 3.22 (2.95, 3.49)^*^ |
| Gender (ref=male) | | | | | |
| Female | 2.22 (1.48, 2.96)^*^ | 2.20 (1.47, 2.93)^*^ | 2.20 (1.47, 2.94)^*^ | 2.20 (1.47, 2.94)^*^ | 2.18 (1.86, 2.50)^*^ |
| Other | 6.00 (2.72, 9.28)^*^ | 5.84 (2.64, 9.04)^*^ | 5.85 (2.65, 9.06)^*^ | 5.85 (2.64, 9.06)^*^ | 5.63 (5.09, 6.16)^*^ |
| Prefer not to say | 1.09 (-0.02, 2.21) | 1.07 (-0.02, 2.16) | 1.06 (-0.03, 2.15) | 1.06 (-0.03, 2.15) | 1.08 (0.07, 2.09) |
| Sexual orientation (ref=heterosexual) | | | | | |
| Sexuality diverse | 3.53 (2.35, 4.72)^*^ | 3.53 (2.34, 4.72)^*^ | 3.53 (2.35, 4.72)^*^ | 3.53 (2.35, 4.72)^*^ | 3.46 (3.13, 3.79)^*^ |
| Unsure | 1.42 (0.73, 2.12) | 1.41 (0.72, 2.10) | 1.40 (0.72, 2.09) | 1.40 (0.72, 2.09) | 1.40 (0.91, 1.88) |
| Prefer not to say | 2.23 (0.89, 3.58) | 2.21 (0.88, 3.53) | 2.25 (0.90, 3.61) | 2.25 (0.90, 3.61) | 2.20 (1.61, 2.79) |
| Perceived household wealth (ref=high) | | | | | |
| Low | 1.38 (0.98, 1.78) | 1.38 (0.98, 1.78) | 1.35 (0.96, 1.75) | 1.36 (0.96, 1.75) | 1.36 (1.07, 1.65) |
| Prefer not to say | 1.15 (0.64, 1.67) | 1.16 (0.64, 1.69) | 1.15 (0.64, 1.67) | 1.15 (0.64, 1.67) | 1.16 (0.72, 1.60) |
| Language spoken most at home (ref=English) | 1.20 (0.54, 1.86) | 1.18 (0.53, 1.83) | 1.20 (0.54, 1.85) | 1.20 (0.54, 1.85) | 1.18 (0.64, 1.72) |
| COVID-19 diagnosis/quarantine (ref=no) |  | 1.35 (0.88, 1.81) | 1.30 (0.85, 1.75) | 1.30 (0.81, 1.79) | 1.30 (0.96, 1.64) |
| Bushfire personal harm (ref=no) |  |  | 1.95 (1.12, 2.79)^*^ | 1.94 (0.98, 2.90) | 1.98 (1.57, 2.39)^*^ |
| COVID-19 diagnosis/quarantine × bushfire personal harm |  |  |  | 1.02 (0.04, 2.01) |  |
| Intercept | 0.002 (0.001, 0.004)^*^ | 0.002 (0.001, 0.004)^*^ | 0.002 (0.001, 0.004)^*^ | 0.002 (0.001, 0.004)^*^ | 0.002 (-0.62, 0.63)^*^ |
| Random Effects |  |  |  |  |  |
| *N* School | 122 | 122 | 122 | 122 |  |
| σ^2^ | 3.29 | 3.29 | 3.29 | 3.29 |  |
| *SD* | 0.42 | 0.42 | 0.39 | 0.39 |  |
| ICC (95% CI) | 0.15 (0.008,  0.25) | 0.15 (0.03, 0.26) | 0.13 (0.001, 0.22) | 0.13 (0.004, 0.23) |  |
| Model Statistics |  |  |  |  |  |
| Observations | 5,163 | 5,163 | 5,163 | 5,163 | 5,163 |
| Log Likelihood | -842.87 | -841.48 | -837.25 | -837.25 | -839.31 |
| AIC | 1,711.75 | 1,710.97 | 1,704.50 | 1,706.50 | 1,706.62 |
| Marginal R^2^/Conditional R^2^ | .34/.37 | .34/.37 | .34/.37 | .34/.37 |  |
| LRT |  | *χ2*(1)=2.78, *p*=.10 | *χ2*(1)=8.47, *p*=.004 | *χ2*(1)=0.002, *p*=.96 | *χ2*(1)=4.12, *p*<.05 |

*Note:* OR=odds ratio (conditional on fixed effects); 95% CI=95% confidence intervals; ref=reference category, ICC=intraclass correlation coefficient (95% CI estimated via bootstrapped simulation); AIC=akaike information criterion; LRT=likelihood ratio test (Model 2 vs Model 3; Model 3 vs Model 4; Model 4 vs Model 5; Model 5 vs Model 2). Model 1=personal risk factors (fixed effects) + school (random intercepts); Model 2=personal risk factors + COVID-19 diagnosis/quarantine (fixed effects) + school (random intercepts); Model 3=personal risk factors + COVID-19 diagnosis/quarantine + bushfire personal harm (fixed effects) + school (random intercepts); Model 4=personal risk factors + COVID-19 diagnosis/quarantine + bushfire personal harm + COVID-19 diagnosis/quarantine × bushfire personal harm (fixed effects) + school (random intercepts); Model 5=personal risk factors + COVID-19 diagnosis/quarantine (fixed effects). Model 3 identified as best fitting model. ^*^*p*<.007.

**Table S11**

*Binomial models predicting elevated COVID-19 trauma from personal risk factors, COVID-19 diagnosis/quarantine, bushfire personal harm, and COVID-19 diagnosis/quarantine × bushfire personal harm. Main effects and interactions between disaster variables were added sequentially in Models 2, 3 and 4. Model 5 is the fixed effects model of the best fitting model (Model 2)*

| Explanatory Variables | Mixed Effects Models | | | | Fixed Effects Model |
| --- | --- | --- | --- | --- | --- |
|  | Model 1 | Model 2 | Model 3 | Model 4 | Model 5 |
| Fixed effects, OR (95% CI) | | | | | |
| Adverse childhood experiences (ref=no) | 2.13 (1.74, 2.52)^*^ | 2.09 (1.70, 2.47)^*^ | 2.08 (1.70, 2.47)^*^ | 2.08 (1.70, 2.47)^*^ | 2.08 (1.90, 2.26)^*^ |
| Mental illness history (ref=no) | 1.79 (1.49, 2.09)^*^ | 1.77 (1.47, 2.06)^*^ | 1.76 (1.46, 2.06)^*^ | 1.76 (1.46, 2.06)^*^ | 1.76 (1.59, 1.93)^*^ |
| Gender (ref=male) | | | | | |
| Female | 2.44 (2.04, 2.85)^*^ | 2.44 (2.03, 2.84)^*^ | 2.44 (2.03, 2.84)^*^ | 2.44 (2.03, 2.84)^*^ | 2.44 (2.27, 2.60)^*^ |
| Other | 3.20 (2.00, 4.39)^*^ | 3.10 (1.94, 4.27)^*^ | 3.09 (1.93, 4.25)^*^ | 3.09 (1.93, 4.25)^*^ | 3.09 (2.72, 3.46)^*^ |
| Prefer not to say | 2.02 (1.01, 3.04)^*^ | 2.00 (0.99, 3.00) | 2.00 (1.00, 3.00) | 2.00 (0.99, 3.00) | 2.00 (1.50, 2.50) |
| Sexual orientation (ref=heterosexual) | | | | | |
| Sexuality diverse | 1.97 (1.56, 2.37)^*^ | 1.96 (1.56, 2.37)^*^ | 1.97 (1.56, 2.37)^*^ | 1.97 (1.56, 2.37)^*^ | 1.97 (1.76, 2.17)^*^ |
| Unsure | 1.09 (0.82, 1.37) | 1.08 (0.81, 1.36) | 1.08 (0.81, 1.36) | 1.08 (0.81, 1.36) | 1.08 (0.83, 1.34) |
| Prefer not to say | 1.50 (1.03, 1.98) | 1.48 (1.01, 1.95) | 1.48 (1.01, 1.95) | 1.48 (1.01, 1.95) | 1.48 (1.17, 1.80) |
| Perceived household wealth (ref=high) | | | | | |
| Low | 1.29 (1.09, 1.49)^*^ | 1.29 (1.09, 1.49)^*^ | 1.29 (1.08, 1.49)^*^ | 1.29 (1.08, 1.49)^*^ | 1.29 (1.13, 1.44)^*^ |
| Prefer not to say | 1.15 (0.89, 1.41) | 1.17 (0.90, 1.43) | 1.16 (0.90, 1.43) | 1.16 (0.90, 1.43) | 1.16 (0.94, 1.39) |
| Language spoken most at home (ref=English) | 1.27 (0.91, 1.63) | 1.25 (0.89, 1.60) | 1.25 (0.90, 1.60) | 1.25 (0.90, 1.60) | 1.25 (0.97, 1.53) |
| COVID-19 diagnosis/quarantine (ref=no) |  | 1.55 (1.26, 1.84)^*^ | 1.53 (1.24, 1.82)^*^ | 1.53 (1.23, 1.84)^*^ | 1.53 (1.35, 1.72)^*^ |
| Bushfire personal harm (ref=no) |  |  | 1.19 (0.87, 1.52) | 1.19 (0.82, 1.57) | 1.19 (0.93, 1.46) |
| COVID-19 diagnosis/quarantine × bushfire personal harm |  |  |  | 1.00 (0.39, 1.61) |  |
| Intercept | 0.05 (0.04, 0.06)^*^ | 0.04 (0.03, 0.05)^*^ | 0.04 (0.03, 0.05)^*^ | 0.04 (0.03, 0.05)^*^ | 0.04 (-0.17, 0.26)^*^ |
| Random Effects |  |  |  |  |  |
| *N* School | 122 | 122 | 122 | 122 |  |
| σ^2^ | 3.29 | 3.29 | 3.29 | 3.29 |  |
| *SD* | 0.20 | 0.19 | 0.19 | 0.19 |  |
| ICC (95% CI) | 0.04 (6.96e^-11^,  8.30e^-02^) | 0.04 (1.03e^-09^,  7.80e^-02^) | 0.04 (5.42e^-13^,  7.89^-02^) | 0.04 (0.0001, 0.10) |  |
| Model Statistics |  |  |  |  |  |
| Observations | 5,600 | 5,600 | 5,600 | 5,600 | 5,600 |
| Log Likelihood | -2,419.60 | -2,409.74 | -2,408.94 | -2,408.94 | -2,410.53 |
| AIC | 4,865.20 | 4,847.48 | 4,847.87 | 4,849.87 | 4,849.05 |
| Marginal R^2^/Conditional R^2^ | .15/.16 | .16/.16 | .16/.17 | .16/.17 |  |
| LRT |  | *χ2*(1)=19.71, *p*<.001 | *χ2*(1)=1.61, *p*=.20 | *χ2*(1)=0.0001, *p*=.99 | *χ2*(1)=1.57, *p=*.21 |

*Note:* OR=odds ratio (conditional on fixed effects); 95% CI=95% confidence intervals; ref=reference category, ICC=intraclass correlation coefficient (95% CI estimated via bootstrapped simulation); AIC=akaike information criterion; LRT=likelihood ratio test (Model 2 vs Model 3; Model 3 vs Model 4; Model 4 vs Model 5; Model 5 vs Model 2). Model 1=personal risk factors (fixed effects) + school (random intercepts); Model 2=personal risk factors + COVID-19 diagnosis/quarantine (fixed effects) + school (random intercepts); Model 3=personal risk factors + COVID-19 diagnosis/quarantine + bushfire personal harm (fixed effects) + school (random intercepts); Model 4=personal risk factors + COVID-19 diagnosis/quarantine + bushfire personal harm + COVID-19 diagnosis/quarantine × bushfire personal harm (fixed effects) + school (random intercepts); Model 5=personal risk factors + COVID-19 diagnosis/quarantine (fixed effects). Model 2 identified as best fitting model. ^*^*p*<.007.

**Table S12**

*Binomial models predicting elevated bushfire trauma from personal risk factors, COVID-19 diagnosis/quarantine, bushfire personal harm, and COVID-19 diagnosis/quarantine × bushfire personal harm. Main effects and interactions between disaster variables were added sequentially in Models 2, 3 and 4. Model 5 is the fixed effects model of the best fitting model (Model 3)*

| Explanatory Variables | Mixed Effects Models | | | | Fixed Effects Model |
| --- | --- | --- | --- | --- | --- |
|  | Model 1 | Model 2 | Model 3 | Model 4 | Model 5 |
| Fixed effects, OR (95% CI) | | | | | |
| Adverse childhood experiences (ref=no) | 1.55 (0.86, 2.24) | 1.47 (0.81, 2.14) | 1.45 (0.80, 2.11) | 1.46 (0.80, 2.12) | 1.45 (1.00, 1.90) |
| Mental illness history (ref=no) | 1.67 (1.02, 2.31) | 1.63 (1.00, 2.26) | 1.58 (0.96, 2.20) | 1.60 (0.97, 2.23) | 1.58 (1.19, 1.97) |
| Gender (ref=male) | | | | | |
| Female | 2.03 (1.27, 2.78)^*^ | 1.99 (1.25, 2.74)^*^ | 2.07 (1.29, 2.85)^*^ | 2.08 (1.29, 2.87)^*^ | 2.08 (1.71, 2.46)^*^ |
| Other | 1.69 (0.21, 3.18) | 1.50 (0.17, 2.84) | 1.48 (0.15, 2.81) | 1.47 (0.14, 2.79) | 1.47 (0.57, 2.36) |
| Prefer not to say | 2.68 (-0.68, 6.04) | 2.67 (-0.69, 6.04) | 2.88 (-0.77, 6.53) | 2.79 (-0.76, 6.34) | 2.90 (1.64, 4.17) |
| Sexual orientation (ref=heterosexual) | | | | | |
| Sexuality diverse | 2.89 (1.43, 4.36)^*^ | 2.96 (1.46, 4.46)^*^ | 3.06 (1.49, 4.63)^*^ | 3.07 (1.50, 4.64)^*^ | 3.07 (2.56, 3.58)^*^ |
| Unsure | 1.18 (0.48, 1.88) | 1.15 (0.46, 1.84) | 1.18 (0.47, 1.89) | 1.21 (0.48, 1.94) | 1.18 (0.58, 1.78) |
| Prefer not to say | 1.90 (0.49, 3.32) | 1.81 (0.46, 3.17) | 1.87 (0.45, 3.29) | 1.85 (0.44, 3.26) | 1.87 (1.12, 2.63) |
| Perceived household wealth (ref=high) | | | | | |
| Low | 1.23 (0.77, 1.69) | 1.23 (0.77, 1.69) | 1.18 (0.73, 1.62) | 1.19 (0.74, 1.65) | 1.17 (0.80, 1.55) |
| Prefer not to say | 1.43 (0.70, 2.16) | 1.44 (0.70, 2.18) | 1.43 (0.69, 2.17) | 1.45 (0.70, 2.20) | 1.43 (0.91, 1.94) |
| Language spoken most at home (ref=English) | 1.30 (0.23, 2.36) | 1.24- (0.21, 2.27) | 1.22 (0.21, 2.23) | 1.23 (0.21, 2.24) | 1.21 (0.39, 2.04) |
| COVID-19 diagnosis/quarantine (ref=no) |  | 1.56 (0.91, 2.22) | 1.40 (0.81, 2.00) | 1.06 (0.31, 1.81) | 1.40 (0.98, 1.83) |
| Bushfire personal harm (ref=no) |  |  | 2.36 (1.54, 3.18)^*^ | 2.15 (1.32, 2.99)^*^ | 2.35 (2.01, 2.70)^*^ |
| COVID-19 diagnosis/quarantine × bushfire personal harm |  |  |  | 1.56 (0.18, 2.95) |  |
| Intercept | 0.08 (0.04, 0.12)^*^ | 0.08 (0.04, 0.11)^*^ | 0.05 (0.02, 0.08)^*^ | 0.05 (0.02, 0.08)^*^ | 0.05 (-0.50, 0.60)^*^ |
| Random Effects |  |  |  |  |  |
| *N* School | 105 | 105 | 105 | 105 |  |
| σ^2^ | 3.29 | 3.29 | 3.29 | 3.29 |  |
| *SD* | 0.22 | 0.21 | 0.14 | 0.13 |  |
| ICC (95% CI) | 0.04 (0.00, 0.17) | 0.04 (0.00, 0.19) | 0.02 (0.00, 0.24) | 0.02 (0.00, 0.15) |  |
| Model Statistics |  |  |  |  |  |
| Observations | 874 | 874 | 874 | 874 | 874 |
| Log Likelihood | -426.19 | -424.09 | -412.10 | -411.60 | -412.13 |
| AIC | 878.39 | 876.18 | 854.20 | 855.20 | 852.25 |
| Marginal R^2^/Conditional R^2^ | .12/.13 | .13/.14 | .18/.19 | .18/.18 |  |
| LRT |  | *χ2*(1)=4.21, *p*=.04 | *χ2*(1)=23.98, *p*<.001 | *χ2*(1)=1.01, *p*=.32 | *χ2*(1)=0.05, *p*=.82 |

*Note:* OR=odds ratio (conditional on fixed effects); 95% CI=95% confidence intervals; ref=reference category, ICC=intraclass correlation coefficient (95% CI estimated via bootstrapped simulation); AIC=akaike information criterion; LRT=likelihood ratio test (Model 2 vs Model 3; Model 3 vs Model 4; Model 4 vs Model 5; Model 5 vs Model 2). Model 1=personal risk factors (fixed effects) + school (random intercepts); Model 2=personal risk factors + COVID-19 diagnosis/quarantine (fixed effects) + school (random intercepts); Model 3=personal risk factors + COVID-19 diagnosis/quarantine + bushfire personal harm (fixed effects) + school (random intercepts); Model 4=personal risk factors + COVID-19 diagnosis/quarantine + bushfire personal harm + COVID-19 diagnosis/quarantine × bushfire personal harm (fixed effects) + school (random intercepts); Model 5=personal risk factors + COVID-19 diagnosis/quarantine (fixed effects). Model 3 identified as best fitting model. ^*^*p*<.007.

**Table S13**

*Likelihood ratio tests, which compare models with and without two-way interactions between personal risk factors (adverse childhood experiences, mental illness history, gender, sexual orientation, perceived household wealth) and disaster (COVID-19 diagnosis/quarantine, bushfire personal harm) for each mental health measure. Comparisons provide a direct test of the stress-diathesis model*

|  | Psychological Distress | Depression | Anxiety | Insomnia | Suicidal Ideation | COVID-19 Trauma | Bushfire Trauma |
| --- | --- | --- | --- | --- | --- | --- | --- |
| Moderator variable |  |  |  |  |  |  |  |
| Adverse childhood  experiences | *χ2*(1)=1.66, *p*=.44 | *χ2*(1)=6.06, *p*=.048^*^ | *χ2*(1)=1.27, *p*=.53 | *χ2*(1)=4.21, *p*=.12 | *χ2*(1)=3.57, *p*=.17 | *χ2*(1)=5.38, *p*=.07 | *χ2*(1)=2.87, *p*=.24 |
| Mental illness history | *χ2*(2)=0.34, *p*=.84 | *χ2*(2)=0.84, *p*=.66 | *χ2*(2)=0.89 *p*=.82 | *χ2*(2)=0.42, *p*=.81 | *χ2*(2)=6.86, *p*=.03^*^ | *χ2*(2)=2.27, *p*=.32 | *χ2*(2)=9.12, *p*=.01^*^ |
| Gender | *χ2*(6)=9.03, *p*=.17 | *χ2*(6)=10.23, *p*=.12 | *χ2*(6)=5.16, *p*=.52 | *χ2*(6)=2.60, *p*=.86 | *χ2*(6)=1.85, *p*=.93 | *χ2*(6)=7.90, *p*=.25 | *χ2*(6)=4.28, *p*=.64 |
| Sexual orientation | *χ2*(6)=8.04, *p*=.24 | *χ2*(6)=2.59, *p*=.86 | *χ2*(6)=6.18, *p*=.40 | *χ2*(6)=5.25, *p*=.51 | *χ2*(6)=2.00, *p*=.92 | *χ2*(6)=2.37, *p*=.88 | *χ2*(6)=10.54, *p*=.10 |
| Perceived household  wealth | *χ2*(4)=4.72, *p*=.32 | *χ2*(4)=4.20, *p*=.38 | *χ2*(4)=5.60, *p*=.23 | *χ2*(4)=3.21, *p*=.52 | *χ2*(4)=5.39, *p*=.25 | *χ2*(4)=3.16, *p*=.53 | *χ2*(4)=3.72, *p*=.45 |

*Note.* Each row represents a new model for each mental health measure. Each of the models were compared to a main effect model including adverse childhood experiences + mental illness history + gender + sexuality + perceived household wealth + language spoken most at home + COVID-19 diagnosis/quarantine + bushfire personal harm (fixed effects) + school (random intercepts). Two-way interactions between disaster (COVID-19 diagnosis/quarantine, bushfire personal harm) and one personal risk factor (e.g., adverse childhood experiences) were then added simultaneously into the models. For example, adverse childhood experiences + mental illness history + gender + sexuality + perceived household wealth + language spoken most at home + COVID-19 diagnosis/quarantine + bushfire personal harm + COVID-19 diagnosis/quarantine × adverse childhood experiences + bushfire personal harm × adverse childhood experiences (fixed effects) + school (random intercepts). ^*^*p*<.05.

**Table S14**

*Predicted probabilities (standard error) for each level of the significant disaster × adverse childhood experiences and disaster × mental illness history interactions presented in Table S1 for depression, suicidal ideation, and bushfire trauma*

| Comparison | | Depression |
| --- | --- | --- |
| COVID-19 diagnosis/quarantine | Adverse childhood experiences |  |
| No | No | 0.10 (0.04) |
| Yes | No | 0.07 (0.04) |
| No | Yes | 0.40 (0.03) |
| Yes | Yes | 0.47 (0.04) |
| Bushfire personal harm | Adverse childhood experiences |  |
| No | No | 0.13 (0.03) |
| Yes | No | 0.06 (0.04) |
| No | Yes | 0.38 (0.03) |
| Yes | Yes | 0.49 (0.05) |
| Comparison |  | Suicidal Ideation |
| COVID-19 diagnosis/quarantine | Mental illness history |  |
| No | No | 0.04 (0.01) |
| Yes | No | 0.06 (0.02) |
| No | Yes | 0.09 (0.03) |
| Yes | Yes | 0.08 (0.03) |
| Bushfire personal harm | Mental illness history |  |
| No | No | 0.03 (0.01) |
| Yes | No | 0.07 (0.02) |
| No | Yes | 0.08 (0.02) |
| Yes | Yes | 0.09 (0.04) |
| Comparison | | Bushfire Trauma |
| COVID-19 diagnosis/quarantine | Mental illness history |  |
| No | No | 0.27 (0.06) |
| Yes | No | 0.25 (0.07) |
| No | Yes | 0.30 (0.08) |
| Yes | Yes | 0.58 (0.01) |
| Bushfire personal harm | Mental illness history |  |
| No | No | 0.17 (0.05) |
| Yes | No | 0.37 (0.08) |
| No | Yes | 0.39 (0.09) |
| Yes | Yes | 0.49 (0.09) |

**Table S15**

*Simple effect comparisons for the significant disaster × adverse childhood experiences and disaster × mental illness history interactions for presented in Table S1 for depression, suicidal ideation, and bushfire trauma*

| Mental Health Measure | Simple Effect Comparisons (COVID-19 Diagnosis/Quarantine) | | OR (95% CI) |
| --- | --- | --- | --- |
| Depression | COVID-19 diagnosis/ quarantine | Adverse childhood experiences |  |
|  | No | No (ref) vs yes | 5.89 (2.77, 12.50)^*^ |
|  | Yes | No (ref) vs yes | 11.07 (3.81, 32.10)^*^ |
|  | Adverse childhood experiences | COVID-19 diagnosis/quarantine |  |
|  | No | No (ref) vs yes | 0.70 (0.31, 1.58) |
|  | Yes | No (ref) vs yes | 1.31 (1.04, 1.66) |
| Suicidal ideation | COVID-19 diagnosis/ quarantine | Mental illness history |  |
|  | No | No (ref) vs yes | 2.68 (1.67, 4.29)^*^ |
|  | Yes | No (ref) vs yes | 1.47 (0.72, 2.97) |
|  | Mental illness history | COVID-19 diagnosis/ quarantine |  |
|  | No | No (ref) vs yes | 1.65 (1.06, 2.56) |
|  | Yes | No (ref) vs yes | 0.90 (0.53, 1.55) |
| Bushfire trauma | COVID-19 diagnosis/quarantine | Mental illness history |  |
|  | No | No (ref) vs yes | 1.20 (0.76, 1.91) |
|  | Yes | No (ref) vs yes | 4.10 (1.82, 9.22)^*^ |
|  | Mental illness history | COVID-19 diagnosis/ quarantine |  |
|  | No | No (ref) vs yes | 0.93 (0.55, 1.59) |
|  | Yes | No (ref) vs yes | 3.19 (1.48, 6.88)^*^ |
| Mental Health Measure | Simple Effect Comparisons (Bushfire Personal Harm) | | OR (95% CI) |
| Depression | Bushfire personal harm | Adverse childhood experiences |  |
|  | No | No (ref) vs yes | 4.38 (2.86, 6.73)^*^ |
|  | Yes | No (ref) vs yes | 14.86 (3.28, 67.37)^*^ |
|  | Adverse childhood experiences | Bushfire personal harm |  |
|  | No | No (ref) vs yes | 0.45 (0.10, 1.93) |
|  | Yes | No (ref) vs yes | 1.51 (1.10, 2.06) |
| Suicidal ideation | Bushfire personal harm | Mental illness history |  |
|  | No | No (ref) vs yes | 3.03 (2.10, 4.38)^*^ |
|  | Yes | No (ref) vs yes | 1.30 (0.55, 3.06) |
|  | Mental illness history | Bushfire personal harm |  |
|  | No | No (ref) vs yes | 2.60 (1.55, 4.33)^*^ |
|  | Yes | No (ref) vs yes | 1.11 (0.53, 2.32) |
| Bushfire trauma | Bushfire personal harm | Mental illness history |  |
|  | No | No (ref) vs yes | 3.07 (1.63, 5.79)^*^ |
|  | Yes | No (ref) vs yes | 1.61 (0.89, 2.90) |
|  | Mental illness history | Bushfire personal harm |  |
|  | No | No (ref) vs yes | 2.88 (1.91, 4.32)^*^ |
|  | Yes | No (ref) vs yes | 1.51 (0.76, 2.99) |

*Note.* ^*^*p*<.007.
